# Ethnopharmacological *in vitro* studies on Austria's folk medicine—An unexplored lore *in vitro* anti-inflammatory activities of 71 Austrian traditional herbal drugs^☆^

**DOI:** 10.1016/j.jep.2013.06.007

**Published:** 2013-10-07

**Authors:** Sylvia Vogl, Paolo Picker, Judit Mihaly-Bison, Nanang Fakhrudin, Atanas G. Atanasov, Elke H. Heiss, Christoph Wawrosch, Gottfried Reznicek, Verena M. Dirsch, Johannes Saukel, Brigitte Kopp

**Affiliations:** a Department of Pharmacognosy, University of Vienna, Althanstrasse 14, A-1090 Vienna, Austria; b Department of Vascular Biology and Thrombosis Research, Medical University of Vienna, Schwarzspanierstrasse 17, A-1090 Vienna, Austria

**Keywords:** Traditional European medicine (TEM), Austria, Inflammation, PPAR, NF-ĸB, IL-8, E-selectin

## Abstract

**Ethnopharmacological relevance:**

In Austria, like in most Western countries, knowledge about traditional medicinal plants is becoming scarce. Searching the literature concerning Austria's ethnomedicine reveals its scant scientific exploration.

Aiming to substantiate the potential of medicinal plants traditionally used in Austria, 63 plant species or genera with claimed anti-inflammatory properties listed in the VOLKSMED database were assessed for their *in vitro* anti-inflammatory activity.

**Material and methods:**

71 herbal drugs from 63 plant species or genera were extracted using solvents of varying polarities and subsequently depleted from the bulk constituents, chlorophylls and tannins to avoid possible interferences with the assays. The obtained 257 extracts were assessed for their *in vitro* anti-inflammatory activity. The expression of the inflammatory mediators E-selectin and interleukin-8 (IL-8), induced by the inflammatory stimuli tumor necrosis factor alpha (TNF-α) and the bacterial product lipopolysaccharide (LPS) was measured in endothelial cells. The potential of the extracts to activate the nuclear factors PPARα and PPARγ and to inhibit TNF-α-induced activation of the nuclear factor-kappa B (NF-κB) in HEK293 cells was determined by luciferase reporter gene assays.

**Results:**

In total, extracts from 67 of the 71 assessed herbal drugs revealed anti-inflammatory activity in the applied *in vitro* test systems. Thereby, 30 could downregulate E-selectin or IL-8 gene expression, 28 were strong activators of PPARα or PPARγ (inducing activation of more than 2-fold at a concentration of 10 µg/mL) and 21 evoked a strong inhibition of NF-κB (inhibition of more than 80% at 10 µg/mL).

**Conclusion:**

Our research supports the efficacy of herbal drugs reported in Austrian folk medicine used for ailments associated with inflammatory processes. Hence, an ethnopharmacological screening approach is a useful tool for the discovery of new drug leads.

## Graphical abstract

**Figure:**
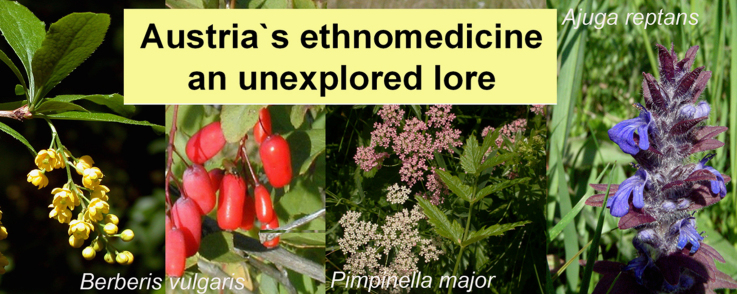

## 1 Introduction

In contrast to traditional Chinese medicine (TCM), traditional European medicine (TEM), including Austria's ethnomedicine, has been barely scientifically explored (Adams et al., 2009). Although the advance of Western medicine and the loss of knowledge in the medical profession and in wide sections of the Austrian population concerning TEM led to repression of the use of traditional preparations, Austria and its adjacent regions have a rich history in traditional folk medicine. In order to conserve this knowledge, the so-called VOLKSMED database was generated by gathering information about traditionally used medicines in Austria and the immediate vicinity (Gerlach et al., 2006; Saric-Kundalic et al., 2010). It refers to knowledge that had been passed on from generation to generation and does not lean on any existing literature. Besides an exact identification of the medicinal product, it contains information about the plant/animal parts or products used, preparation and application methods, internal or external use, and indications. The whole database comprises 43,150 entries, and is based on 67,200 interviews. Regarding inflammatory ailments, the database includes 8457 statements on 226 preparations of 123 plant species or genera and 24 animal products. The VOLKSMED database comprises approximately 700 plant and fungi species/genera, however, only 196 are mentioned more than 50 times. Hence, this database was the basis for the selection of herbal drugs to be evaluated on various anti-inflammatory targets, using *in vitro* cell based assays.

Inflammation is part of various pathological conditions *e.g.* arthritis, atherosclerosis, the metabolic syndrome, allergies and other autoimmune diseases as well as cancer. For most of these conditions no satisfying treatment of the associated inflammation is available. General treatment relies on steroidal and non-steroidal anti-inflammatory drugs (NSAIDs) which have several adverse effects (McGettigan et al. 2011) as well as on disease-modifying anti-rheumatic drugs (DMARDs). Therefore, the search for new anti-inflammatory drugs still represents an important field in drug discovery.

Many inflammatory processes in different cell types are mediated by the transcription factor nuclear factor kappa-light-chain-enhancer of activated B-cells (NF-κB). Major target genes of NF-κB include those for transcription of some pro-inflammatory adhesion molecules (*e.g.*, E-selectin), cytokines (*e.g.*, tumor necrosis factor (TNF)-α), growth factors and enzymes (*e.g.*, cyclooxygenase (COX)-2) producing inflammatory mediators. Among the most prominent mediators of inflammation are cytokines such as TNF-α and interleukin (IL)-1, as well as components released by bacteria such as LPS. Important molecular players inhibiting NF-κB responses are nuclear receptors, like the glucocorticoid receptor, the peroxisome proliferator-activated receptors (PPARs), the farnesoid X receptor (FXR), the liver X receptor (LXR) and the orphan nuclear receptors class 4A (NR4A). Nuclear receptors are thought to exert their anti-inflammatory activities by repressing the activity of other transcription factors, such as NF-κB, STAT or AP1 proteins, which are involved in the induction of a variety of pro-inflammatory genes (Karin et al., 2004; Glass and Ogawa, 2006; Bamborough et al., 2009; Brasier, 2010).

In the present study, a panel of functional as well as target-oriented cell models were used for the identification of active natural products. Thereby, interference with TNF-α or LPS induced expression of the adhesion molecule E-selectin or the chemokine IL-8, inhibition of the NF-κB pathway and activation of nuclear receptors (PPARα and PPARγ) were investigated, in order to get insight in various steps of the complex inflammatory process.

Since there were no data available concerning the interference of bulk constituents, such as chlorophylls and tannins, with the respective test systems, separation of those was implemented. However, due to the possibility of co-separation of active constituents, not only the purified extracts but also the crude extracts were tested for activity.

## 2 Materials and methods

### 2.1 Literature search

In a first step, 123 plant species or genera, reported in the VOLKSMED database as traditionally used anti-inflammatory agents (*e.g.*, for colds, rheumatism, gastro-intestinal diseases), were explored in the databases Chemical Abstracts (CAPLUS) and Medline. The search was limited to terms concerning inflammation (antiphlogistic, anti-inflammatory, *etc.*) in order to avoid re-investigations.

Plant species/genera with insufficient coverage in the literature and a good score in the VOLKSMED database were considered as interesting candidates. Further important points had been the availability of the herbal drugs on the market or the possibility to collect the material by wild harvesting. Using these selection criteria, 63 promising plant species/genera were selected for further investigations.

### 2.2 Plant material

Part of the plant material could be purchased from various suppliers (Kottas Pharma GmbH, Vienna, Austria; Alfred Richter GmbH. & CO. KG, Kufstein, Austria and Alfred Galke GmbH, Gittelde, Germany). Several species have been collected in Austria (Table 1) in most cases at the flowering period. The plant material was authenticated by one of the authors (J. Saukel). Voucher specimens are deposited in the Herbarium of the Institute of Pharmacognosy, University of Vienna (WUP). The material was air dried at room temperature.

### 2.3 Reagents and chemicals

Methanol (MeOH), chloroform (CHCl_3_), hexane and dichloromethane (DCM) were of p.a. grade (VWR, Vienna, Austria). DMSO p.a. for *in vitro* tests was purchased from Carl Roth GmbH und Co. KG, Karlsruhe, Germany. Water was distilled by an automatic distillation apparatus (IKA-Dest M3000, IKA^®^-Werke GmbH & Co. KG, Staufen, Germany).

For the cell based *in vitro* assays, human embryonic kidney (HEK) 293 cells were obtained from the American Type Culture Collection (ATCC, Manassas, VA, USA). HEK293 cells stably transfected with an NF-κB luciferase reporter (HEK293/NF-κB-luc cells) were bought from Panomics (RC0014; Fremont, *CA*, USA). Professor Ronald M. Evans (Howard Hughes Medical Institute, La Jolla, CA, USA) kindly provided the PPAR luciferase reporter construct (tk-PPREx3-luc) and the expression plasmids for PPARα and PPARγ (pCMX-mPPARα and pCMX-mPPARγ). The plasmid encoding enhanced green fluorescent protein (pEGFP-N1) was purchased from Clontech (Mountain View, CA, USA). Fetal bovine serum (FBS) was obtained from Invitrogen (Lofer, Austria) and Dulbecco's modified Eagle's medium (DMEM) containing 4.5 g/L glucose as well as the L-glutamine were from Lonza Group AG (Basel, Switzerland).

### 2.4 Extraction and removal of bulk constituents

The dried plant material was pulverized and extracted with the nonpolar solvent, dichloromethane (DCM), followed by the polar solvent methanol (MeOH). Extraction was performed at 40 °C and 150 bar with 3 extraction cycles, 5 min heat-up time, 2 min static time, 10% flush volume and 60 s nitrogen purge in an accelerated solvent extractor ASE200 (Dionex Austria GmbH, Vienna, Austria).

**Fig. 1:**
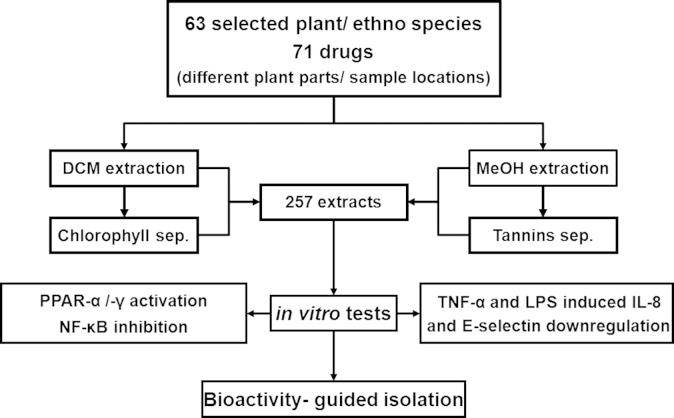
Overview of plant processing and employed test systems.

To exclude possible interferences with the used *in vitro* assays, tannins and chlorophyll were partitioned from polar and nonpolar extracts, respectively (Vogl et al., 2013). The tannins removal method was based on liquid–liquid partitions between CHCl_3_ and mixtures of MeOH/H_2_O (Wall et al., 1996). For the exclusion of chlorophyll from the green plant material, a method based on a liquid–liquid partition between dichloromethane and a mixture of MeOH/H_2_O was used. Briefly, 1 g of dichloromethane extract was dissolved in 150 mL DCM. Further, the same amount of MeOH/H_2_O (1:1) was added. Finally, DCM was removed under reduced pressure leading to precipitation of the chlorophyll, which could easily be filtered. This process leads to a loss of about 50–90% of yield of crude extract. Fig. 1 gives an overview of extraction and purification processes, as well as pharmacological evaluation steps.

Prior to bioactivity evaluation, all extracts were dried, reconstituted in dimethyl sulfoxide (DMSO), aliquoted, and stored at −20 °C until use.

### 2.5 PPAR transactivation assay

The PPAR transactivation assay was performed as previously described (Rozema et al., 2012; Vogl et al., 2013). 6×10^6^ HEK 293 cells were seeded in 10 cm dishes and incubated overnight. On the next day cells were transfected by the calcium phosphate precipitation method with a total of 10 µg DNA including the PPAR luciferase reporter construct (PPRE-tk3x-Luc), the expression plasmid for the corresponding PPAR receptor protein (PPARα or PPARγ) and pEGFP-N1 (Clontech Laboratories, Inc. Mountain View, USA) as internal control. The ratio of the DNA of PPRE:PPAR:EGFP was maintained at 2:2:1. After 6 h cells were seeded into 96-well plates and the medium was substituted with a DMEM supplemented with 5% charcoal stripped FBS. After 1 h incubation the indicated treatments were performed and cells were further incubated at 37 °C for 18 h. Cells were then washed with phosphate buffered saline (PBS) and lysed. The luminescence of the firefly luciferase as well as the fluorescence of EGFP were quantified on a GeniosPro plate reader (Tecan, Grödig, Austria). The luciferase signal was normalized with the EGFP derived fluorescence to account for differences in the cell number and/or transfection efficiency. The specific PPARα agonist GW7647 (Cayman, Missouri, USA) and the PPARγ agonist pioglitazone (Molekula Ltd, Shaftesbury, UK) were used as positive controls.

### 2.6 NF-κB transactivation assay

HEK293 cells stably transfected with the NF-κB-driven luciferase reporter gene NF-κB-luc (293/NF-κB-luc cells, Panomics, RC0014) were seeded in 10 cm dishes and transfected with 5 µg pEGFP-N1 (Clontech Laboratories, Inc. Mountain View, USA). The NF-κB transactivation assay was performed as previously described (Rozema et al., 2012; Vogl et al., 2013). Six hours after transfection the cells were transferred into 96-well plates containing a serum-free DMEM and incubated at 37 °C overnight. On the next morning, cells were pre-treated with the indicated extracts for 1 h. Thereafter, cells were stimulated with 2 ng/mL human recombinant TNF-α for 6 h, then the medium was removed and cells were lysed with luciferase reporter lysis buffer (E3971, Promega, Madison, USA). Plant extracts and reference pure compounds were tested at a concentration of 10 µg/mL in at least three independent experiments. Parthenolide was used as positive control at a concentration of 5 µM. The luminescence of the firefly luciferase and the fluorescence of EGFP were quantified on a GeniosPro (Tecan, Grödig, Austria) plate reader. The luciferase signal derived from the NF-κB reporter was normalized with the EGFP-derived fluorescence to account for differences in the cell number.

### 2.7 TNF-α or LPS-induced IL-8 and E-selectin mRNA expression

Immortalized human vascular endothelial cells (HUVECtert) (Chang et al., 2005) were grown in M199 medium (Sigma-Aldrich, St. Luis, USA) supplemented with 20% fetal bovine serum (FBS; Sigma, Taufkirchen, Germany), endothelial cell growth supplement (Technoclone, Austria) and antibiotics. Experiments were performed in 12-well plates (NUNC, Roskilde, Denmark) in M199 medium containing 1% bovine serum albumin (Applichem, Darmstadt, Germany) and 3% FBS.

The testing was started with pooled crude extracts of 10 herbal drugs at concentrations of 100 µg/mL. The extracts were pooled by chance. Further on, the single extracts of the active pools were individually tested at a concentration of 10 µg/mL. The used stimuli TNF-α and LPS are known to activate distinct but partially overlapping signaling pathways with a documented role in acute and chronic inflammation. Quantification of the IL-8 and E-selectin mRNA expression was performed by RT-qPCR: Monolayers of sub-confluent quiescent HUVECtert cells were first treated with the plant material or inhibitor for 10 min. Subsequently, they were stimulated for 30 min or 4 h with 100 ng/mL TNF-α (PeproTech, Rocky Hill, USA) or LPS (Sigma-Aldrich, St. Luis, USA). QIAzol lysis reagent (Qiagen, Hilden, Germany) was used to extract RNA from the cells. Hence, 900 ng of RNA was reverse transcribed with MulV-RT using Oligo d(T) primers (Applied Biosystems, Carlsbad, USA). Relative expression of the investigated genes was then assessed by RT-qPCR (Roche, Basel, Switzerland). Primers were designed with the reference mRNA sequences of respective genes from the GeneBank (URL: http://www.ncbi.nlm.nih.gov) using PRIMER3 software from the Whitehead Institute for Biomedical Research (Cambridge, USA). For E-selectin 5′-ggtttggtgaggtctgctc-3′(forward) and 5′-tgatctgtcccggaactgc-3′(reverse) was applied and for IL-8 primers 5′-ctcttggcagccttcctgatt-3′(forward) and 5′-tatgcactgacatctaagttctttagca-3′(reverse) was used. Relative quantification of the intended genes was performed by normalization to a housekeeping gene β2-microglobulin using the mathematical model by Pfaffl (Kadl et al., 2002). Results were demonstrated as fold variation compared to the control.

## 3 Results and discussion

As the survey on the VOLKSMED database for indications related to inflammation revealed the huge number of 8457 citations, the most promising 123 herbal drugs were chosen. After a literature research concerning the investigation status of these herbal drugs with special emphasis on the planned *in vitro* test systems, 63 plant species/genera from 28 families were selected for phytochemical and pharmacological assessment.

Table 1 reveals information stored in our database concerning the selected plant species/genera, the used plant part(s), the traditional indications and application methods. Further on, the table gives information about the investigated plant material, the location of the sample collection or commercial source of the assessed drug.

The most common internal applications are teas, tinctures and syrups; external applications include baths, washes with teas and tinctures, ointments and the use of crude plant material (*e.g. Euphrasia* sp., *Equisetum* sp., *Beta* sp.). With 9 selected species, the Lamiaceae is the most frequent used family in this study. Most common application forms were teas prepared from the herb. Rosaceae with 8 and Apiaceae with 5 investigated species were also highly represented. From Rosaceae species the herb, fruits and roots were used mainly as tea; fruits were also prepared as jellies and syrups. From the Apiaceae people traditionally used the roots with the most common beverages being teas, liqueurs and wines.

### 3.1 Cell based *in vitro* anti-inflammatory activity

The study at hand presents new *in vitro* screening results concerning anti-inflammatory targets from the following species: *Agropyron repens, Ajuga genevensis, Angelica sylvestris, Bellis perennis, Circaea lutetiana*, *Epilobium montanum, Equisetum palustre, Filipendula vulgaris, Gentiana punctata, Geum montanum, Geum urbanum, Hypericum maculatum, Majorana hortensis, Melampyrum pratense, Pimpinella major, Rumex alpinus and Sorbus aucuparia*.

All results of the *in vitro* cell-based assays regarding PPARα and PPARγ activation, NF-κB inhibition and downregulation of TNF-α- or LPS-induced expression of IL-8 or E-selectin mRNA are summarized in Table 2. For the latter assay some results are missing since initially pools of 10 plants were screened and only the plants from the most active pools were individually tested. The results are shown together with the species or genus name, the plant part used and the extracts thereof. Extracts were tested in triplicate at a concentration of 10 µg/mL on PPARs activation as well as on NF-κB inhibition and at 100 µg/mL concerning LPS- or TNF-α-induced downregulation of interleukine-8 and E-selectin mRNA. An inhibition/activation <25% was considered as no activity, 25–50% inhibition/activation was interpreted as a weak or low activity, 50–75% inhibition/activation was considered as a moderate activity and strong or high activity was defined as >75–100% inhibition/activation.

Extracts from 67 of 71 herbal drugs were active in at least one of the pharmacological test systems. 30 samples (42%) were able to reduce the inflammatory mediators IL-8 and E-selectin. 28 herbal drugs (39%) showed a strong activation of PPARα and/or PPARγ (activation of more than 2-fold at a concentration of 10 µg/mL). Strong inhibition of NF-κB (inhibition of more than 80% at 10 µg/mL) was observed for 21 of the investigated herbal drugs (30%). Surprisingly, the nonpolar dichloromethane extracts gave most hits with 51% strong and 75% moderately active extracts. From the polar methanolic extracts 34% and 37% showed strong and moderate activities, respectively. After removing chlorophylls or tannins, 48% and 35% showed strong and 70% and 61% moderate activity.

This striking hit rate of 95% actives argues for the high effectivity of the ethnopharmacological screening approach compared to random screening with a reported average hit rate of 10% (Harvey, 2002). Moreover, it finally provides some scientific evidence for the traditional usage of several Austrian medicinal plants.

To get deeper insight into the anti-inflammatory action of each investigated plant species results were compared with already published data. The investigated species are listed alphabetically in the following pages.

#### 3.1.1 Agrimonia eupatoria

In the present study an NF-κB inhibition by the crude and the chlorophyll depleted DCM extract of *Agrimonia eupatoria* was observed. Recently, Yoon et al. (2012) reported that in chronic ethanol-fed rats *Agrimonia eupatoria* (30 mg/kg) prevented the ethanol-induced increase in serum concentrations of TNF-α and IL-6, attenuated an increase in p65 level, a subunit of NF-κB, as well as cytochrome CYP2E1 activity, toll like receptor (TLR4) protein expression and TNF-α, IL-6, COX-2, and inducible nitric oxide synthase (iNOS) mRNA (Yoon et al., 2012). Possible active substances could be the flavan-3-ols, flavonols, flavones, procyanidines and phenolic acids identified for *Agrimonia eupatoria* (Correia et al., 2007). Moreover, *Agrimonia eupatoria* herb was found to suppress LPS induced nitric oxide production in BV2 microglial cells as well as the production of pro-inflammatory cytokines such as TNF-α, IL-1β and IL-6 in a dose-dependent manner (Lee et al., 2005). Significant scavenging capacity of reactive oxygen species (ROS) by its polyphenols (Correia et al., 2007), anti-oxidant capacities (Correia et al., 2006) as well as an inhibition of LPS-induced production of nitric oxide and pro-inflammatory cytokines in microglial cells (Bae et al., 2010) have also been reported for this species.

#### 3.1.2 *Ajuga reptans* and *Ajuga genevensis*

In the present study, a downregulation of expression of the pro-inflammatory mediators E-selectin and IL-8 was achieved by the extracts of *Ajuga reptans* and *Ajuga genevensis* herb, supporting already reported activities. An *Ajuga reptans* extract standardized for 50% phenylpropanoid content was tested for anti-inflammatory and antioxidant activity in mice (Marzani et al., 2009). Furthermore, the phenylpropanoid glycoside, teupolioside was shown to reduce pro-inflammatory cytokine release, the appearance of nitrotyrosine and poly(ADP-ribose) polymerase immunoreactivity in the colon of rats, along with an up-regulation of the intercellular adhesion molecule (ICAM-1) and P-selectin. Teupolioside also decreased pro-MMP (Matrixmetalloproteinase) -9 and -2 activity, induced in the colon of rats by dinitrobenzene sulfonic acid administration (Di Paola et al., 2009). Korkina et al. (2007) reported a significantly accelerated wound healing and anti-inflammatory action in the excision wound model, an inhibition of ROS release from the whole blood leukocytes and a ferrous ion chelating capacity for this compound. Moreover, teupolioside inhibited chemokine and growth factor expression by cultured human keratinocytes treated with pro-inflammatory cytokines, TNF-α and interferon-gamma (IFN-γ) (Korkina et al., 2007).

#### 3.1.3 Alnus viridis

Concerning *Alnus viridis,* PPAR activating or NF-κB inhibiting properties have not been reported so far. A possible connection to the observed effects might be the diarylheptanoid derivate oregonin, mainly found in *Alnus* species, which was reported to display anti-inflammatory and anti-oxidative activities (Kuo et al., 2008).

#### 3.1.4 *Angelica archangelica* and *Angelica sylvestris*

In the present study, a moderate NF-κB inhibiting activity was observed for the DCM root extract of *Angelica archangelica,* while a strong one as well as a strong PPAR activation was demonstrated for the tannin free extract of *Angelica sylvestris*. Coumarins isolated from *Angelica archangelica* roots have already been investigated for their anti-inflammatory activity in terms of *in vitro* cyclooxygenase-1 (COX-1) and 5-lipoxygenase (5-LOX) inhibition. Thereby, no activity was found in the COX-1 assay; however, osthole and oxypeucedanin hydrate isovalerate could inhibit 5-LOX (Roos et al., 1997).

#### 3.1.5 Argentina anserina

To the best of our knowledge, a direct effect of *Argentina anserina* extracts on NF-κB, or PPARs, has not been demonstrated so far. A methanolic extract from the herb of *Argentina anserina* (former *Potentilla anserina*) was claimed to inhibit TNF-α-induced cell damage of L-929 cells as measured by the MTT assay, which might include the possibility that inflammatory pathways downstream of the TNF-α receptor, like NF-κB or PPARs, might also be affected (Muraoka et al., 2011).

#### 3.1.6 Berberis vulgaris

The detannified extract of *Berberis vulgaris* fruits showed moderate activity on PPARα. Most publications of this species deal with the alkaloid berberine, isolated from its roots. Cui et al. (2009) demonstrated that this compound was able to decrease T helper 17 and 1 cell cytokine secretion and lowered the activity of the transcription factors Signal Transducers and Activators of Transcription 1 and 4 (STAT1 and STAT4) through the suppression of p38 mitogen-activated protein kinase (MAPK) and c-Jun N-terminal kinase (JNK) activation in type 1 diabetic non-obese diabetic mice (Cui et al., 2009). An anti-inflammatory property of another alkaloid from this species, berbamine, was also indicated via a selective down-regulation of STAT4 as well as by an inhibition of IFN-γ in experimental autoimmune encephalomyelitis (Ren et al., 2008). Moreover, cannabisin G and (±)-lyoniresinol isolated from the root bark of *Berberis vulgaris* exhibited antioxidant activity with IC_50_ values of 2.7 and 1.4 μg/mL in a hydroxyl radical scavenging assay (Tomosaka et al., 2007).

#### 3.1.7 Betonica officinalis

Concerning *Betonica officinalis*, no reports could be found about an activity on the suppression of the chemokine IL-8 or the pro-inflammatory cell adhesion molecule E-selectin assessed in the present study. An antioxidant activity was observed by the total flavonoids isolated from *Betonica officinalis* leaves and roots (Hajdari et al., 2010). In the context of inflammation rosmarinic acid was identified in the aerial parts of *Betonica officinalis* (Czigle et al., 2007).

#### 3.1.8 Calluna vulgaris

In the present study, an inhibition of NF-κB by extracts of *Calluna vulgaris* was described for the first time. In literature, extracts from its aerial parts were investigated for their anti-inflammatory and anti-nociceptive activities *in vivo* using mice models. Here, the flavonol kaempferol-3-*O*-β-D-galactoside was isolated as the active principle (Orhan et al., 2007a). Ursolic acid, identified in heather flowers, was found to inhibit LOX and COX and to block macrophage arachidonic acid metabolism in mouse peritoneal macrophages (Najid et al., 1992). In a screening of Swedish traditional remedies *Calluna vulgaris* and *Geum urbanum* were reported to inhibit prostaglandin biosynthesis and platelet activating factor (PAF)-induced exocytosis *in vitro*. Moreover, *Calluna vulgaris* was identified as one of the most potent COX inhibitors (Tunon et al., 1995).

#### 3.1.9 Capsella bursa-pastoris

All extracts prepared from *Capsella bursa-pastoris* revealed one or the other anti-inflammatory activity in our assays. Yue et al. (2007) studied anti-inflammatory astringent effects of this plant species and found an inhibitory activity on auricular edema, exudation of abdominal blood capillaries and cotton pellet-induced auricle edema.

#### 3.1.10 Epilobium angustifolium, Epilobium montanum and Epilobium parviflorum

Results of the present screening revealed only a moderate potency of *Epilobium angustifolium* and *Epilobium parviflorum* extracts to inhibit NF-κB. However, the less investigated species *Epilobium montanum* also activated PPARs and reduced the expression of IL-8 and E-selectin mRNA after stimulation with TNF-α and LPS. In literature, anti-inflammatory and anti-oxidant activities of *Epilobium angustifolium* and *Epilobium parviflorum* have been linked to their content of oenothein B, quercetin-3-*O*-glucuronide and myricetin-3-*O*-rhamnoside. Extracts from the herb of these two *Epilobium* species suppressed the activity of hyaluronidase and lipoxygenase with IC_50_s around 5 μg/mL and 25 μg/mL. This inhibition was related to the high content of oenothein B, revealing an IC_50_ of 1.1 μM. Moreover, the extracts as well as the pure compound oenothein B (IC_50_=15.4 μM) were able to decrease the release of myeloperoxidase and reactive oxygen species (ROS) from stimulated neutrophils (Kiss et al., 2011). Oenothein B and valoneic acid dilactone isolated from the herb of *Epilobium angustifolium* L. were investigated for their influence on histone acetyltransferase (HAT) and histone deacetylase (HDAC) activity, which play a role in inflammation. Thereby, oenothein B restored cell viability and reversed the effect of TNF-α on HAT and HDAC activities (Kiss et al., 2012). The flavonoid myricetin 3-*O*-β-D-glucuronide, isolated from the leaves of *Epilobium angustifolium,* exhibited anti-inflammatory activity on carrageenan-induced edema in the rat hind paw and an inhibitory effect on prostaglandin biosynthesis (Hiermann et al., 1991). Juan et al. (1988) showed that the aqueous extracts from both *Epilobium angustifolium* and *Epilobium parviflorum* reduced the *in vitro* release of 6-keto-prostaglandin F1α, PG(prostaglandin)E2, and PGD2 from rabbit ear in a concentration dependent manner (Juan et al., 1988). The ethanolic extract of *Epilobium parviflorum* herb revealed hydroxyl radical scavenging activity, inhibited COX-1 and -2 catalyzed prostaglandin biosynthesis and the growth of *Escherichia coli* (Steenkamp et al., 2006).

#### 3.1.11 *Equisetum arvense* and *Equisetum palustre*

In the present study the apolar extracts from *Equisetum arvense* were potent inhibitors of NF-κB and activators of PPARs. The extracts from its related species *Equisetum palustre*, on the other hand, were more effective on the downregulation of the pro-inflammatory mediators IL-8 and E-selectin. Recently, in a review on the phytochemistry and pharmacological properties of *Equisetum arvense* anti-bacterial, anti-fungal, anti-oxidant, analgesic, anti-inflammatory, anti-diabetic, anti-tumor, cytotoxic and anticonvulsant activities were claimed for this species. Additionally, apigenin, luteolin, equisetumoside A, equisetumoside B and equisetumoside C, nicotine, palustrine and palustrinine were reported as phytochemical compounds (Asgarpanah and Roohi, 2012). Oka et al. (2007) reported that the herb suppressed the production of ROS, superoxide anions and hydroxyl radicals generated in cell-free systems and human neutrophils. Moreover, extracts from the aerial parts of *Equisetum arvense* inhibited carrageenan induced paw edema in rats (Oka et al., 2007).

#### 3.1.12 *Euphrasia* sp. (commercial drug) and *Euphrasia rostkoviana*

Regarding the species from the genus *Euphrasia* in our screening*, Euphrasia sp*. (commercial drug) inhibited NF-κB, whereas, *Euphrasia rostkoviana* was additionally active on PPARs. Xu et al. (2008) detected highest antioxidant (ROS) activity in a phenolic acid fraction of *Euphrasia officinalis* herb, followed by its flavonoids. Lowest activity was shown by its iridoid glycosides. Yet, the flavonoid fraction was the most potent inhibitor of NO production in LPS stimulated RAW 264.7 macrophages (Xu et al., 2008).

#### 3.1.13 *Filipendula ulmaria* and *Filipendula vulgaris*

We could detect an activation of PPARs by *Filipendula ulmaria* flowers and an inhibition of NF-κB by the herb. The flowers of *Filipendula vulgaris*, on the other hand, attenuated the expression of IL-8 and E-selectin mRNA. The flowers of *Filipendula ulmaria* were reported to possess complement inhibitory activity (Halkes et al., 1997), as well as to decrease the synthesis of IL-2 by splenocytes and to suppress the production of pro-inflammatory cytokines in delayed-type hypersensitivity reaction (Churin et al., 2008).

#### 3.1.14 Glechoma hederacea

In the present study, extracts from *Glechoma hederacea* were not just able to inhibit NF-κB but did also exert strong activation of PPARs. Recently, rosmarinic acid analogs obtained from the herb of *Glechoma hederacea var. longituba* were shown to inhibit NF-κB production (in a luciferase assay) and the induction of COX-2 and iNOS mRNA in HepG2 cells (Kim et al., 2011). Moreover, this plant species was reported to inhibit IFN-γ and LPS-induced NO production in a dose-dependent manner, as well as the production of the pro-inflammatory cytokines, IL-12p70 and TNF-α in mouse peritoneal macrophages. However, it increased IFN-γ/LPS-induced IL-12p40 production and did not affect IL-6 production (An et al., 2006).

#### 3.1.15 Hippophae rhamnoides

In contrast to our study, investigating the fruits of *Hippophae rhamnoides*, most studies deal with the leaves of this plant species. Polyphenols, polyphenolcarboxylic acids, flavones and flavone derivatives as well as carotene derivatives, isolated from the berries, have been related to its antioxidant and free radical scavenging activities (Popescu et al., 2011). Kwon (2011) isolated casuarinin from the leaves of *Hippophae rhamnoides* and studied its effect on the TNF-α-induced ICAM-1 expression in a human keratinocytes cell line HaCaT. Pre-treatment with casuarinin inhibited TNF-α-induced protein and mRNA expression of ICAM-1 as well as TNF-α-induced NF-κB activation. Furthermore, the activation of extracellular-signal regulated kinase (ERK) and p38 MAPK was suppressed in a dose-dependent manner and TNF-α-induced pro-inflammatory mediators, such as IL-1β, IL-6, IL-8, and monocyte chemoattractant protein-1 (MCP-1) could be decreased after pre-treatment with casuarinin (Kwon et al., 2011). The phenolic compounds (+)-catechin, (+)-gallocatechin, and (−)-epigallocatechin and the tritepenoid ursolic acid were isolated from a 70% ethanolic extract of the branches from sea buckthorn and evaluated for their inhibitory effect on 12-*O*-tetradecanoylphorbol-13-acetate (TPA)-induced inflammation (1 μg/ear) in mice. Thereby, (−)-epi-gallocatechin and ursolic acid showed an effect with a 50% ID of 1.7 and 0.2 μmol/ear (Yasukawa et al., 2009). However, in our study no activity was observed by *Hippophae rhamnoides* fruit extracts concerning NF-κB inhibition, PPAR activation or downregulation of IL-8 and E-selectin.

#### 3.1.16 Linum usitatissimum

In the present study, no *in vitro* anti-inflammatory activity was observed by the different linseed (*Linum usitatissimum*) extracts on NF-κB, PPAR, IL-8 and E-selectin. In 2011 Kaithwas et al. reported that the fixed oil of *Linum usitatissimum* (flaxseed/linseed) inhibited PGE2-, leukotriene-, histamine- and bradykinin-induced inflammation as well as arachidonic acid-induced inflammation, suggesting its capacity to inhibit both cyclooxygenase and lipoxygenase pathways of arachidonate metabolism (Kaithwas et al., 2011). In experimental models, *Linum usitatissimum* fixed oil led to a dose-dependent reduction in joint swelling and circulating TNF-α levels in both preventive and curative protocols of arthritis induced by complete Freund's adjuvant (CFA) and lowered TNF-R1 as well as IL-6 proteins in the arthritic paw (Singh et al., 2012). In an *in vivo* study by Farahpour et al. (2011), the wound healing activity of flaxseed oil on an experimentally induced incision wound was evaluated. Therapeutic ointments containing 0.75% and 1.5% oil were applied to wounds of male rats. Eventually, treated animals showed significant reductions of inflammatory cells in the period of re-epithelization (Farahpour et al., 2011). In a clinical trial Bloedon et al. (2008) studied the effects of flaxseed on markers of cardiovascular risk in hypercholesterolemic adults. 26 men and post-menopausal women with pre-study low density lipoprotein cholesterol (LDL-C) were randomized to 40 g/day of ground flaxseed- or wheat-bran containing baked products. Thereby, *Linum usitatissimum* increased serum levels of alpha-linolenic acid (*p*<0.001), significantly lowered LDL-C at 5 weeks (−13%, *p*<0.005), diminished lipoprotein by a net of 14% (*p*=0.02) and reduced homeostatic model assessment of insulin resistance index by 23.7% (*p*=0.03) at 10 weeks compared to wheat. However, it did not show an effect on markers of inflammation (IL-6, Hs-CRP) or oxidative stress (ox LDL, urinary isoprostanes) at any time points (Bloedon et al., 2008). Flaxseed oil was found to suppress oxygen radical production by white blood cells, to prolong bleeding time, and to suppress the serum levels of inflammatory mediators (IL-1β, IL-2, IL-4, IL-6, IL-10, TNF-α, interferon-gamma, C-reactive protein, and serum amyloid A) but did not lower serum lipids (Prasad, 2009). Singh et al. (2008) screened the fixed oil obtained from the seeds of linseed, containing alpha-linolenic acid for an anti-inflammatory activity *in vivo* using carrageenan, leukotriene and arachidonic acid induced paw edema models in rats. Thereby, significant inhibition of paw edema was observed at the highest dose (3 mL/kg) in all models (Singh et al., 2008).

#### 3.1.17 *Lycopodium sp.* (commercial drug)

Inhibition of NF-κB as well as activation of PPARs by various extracts of *Lycopodium sp.* (commercial drug) was observed in the present study. In literature, anti-inflammatory, anti-oxidant and anti-microbial activities were reported for *Lycopodium* species (Orhan et al., 2007b; Namsa Nima et al., 2009).

#### 3.1.18 *Malva neglecta* and *Malva sp.* (*Malvae folium* according to Ph. Eur.)

In our study, extracts from *Malva neglecta* herb and *Malva sp.* leaves (*Malvae folium* according to Ph. Eur.) were effective modulators of PPARs, NF-κB, E-selectin and IL-8. In a review on *Malva sylvestris* its leaves were reported to have potent anti-inflammatory, antioxidant, anti-complementary, anticancer, skin tissue integrity activity and anti-ulcerogenic effect (Gasparetto et al., 2012). Leaves from *Malva sylvestris* showed strong antioxidant properties, including radical-scavenging activity (EC_50_=0.43 mg/mL), reducing lipid peroxidation in liposomes (0.04 mg/mL) and brain cell homogenates (0.09 mg/mL). Responsible for this effect could be the high contents of antioxidants (phenols, flavonoids, carotenoids, tocopherols), unsaturated fatty acids (α-linolenic acid) and minerals (Barros et al., 2010). Pirbalouti et al. (2010) evaluated the wound healing activity of diethyl ether extracts from *Malva sylvestris* flowers at a dose of 200 mg/kg/day in alloxan-induced diabetic rats. The extract-treated diabetic rats showed significant reduction in the wound area compared to the control. Moreover, tissue samples obtained from the 9th and 18th day after treatment revealed increased well organized bands of collagen, more fibroblasts and few inflammatory cells (Pirbalouti et al., 2010).

#### 3.1.19 Melissa officinalis

In the present study, an *in vitro* inhibition of NF-κB and an activation of PPARs by the nonpolar extracts of *Melissa officinalis* leaves were observed. In the context of inflammation, Mencherini et al. (2009) investigated the influence of substances isolated from the fresh leaves and stems of *Melissa officinalis* on expression of pro-inflammatory mediators. Thereby, sulphated triterpenes, ionol derivatives as well as caffeic and rosmarinic acid neither affected cell viability nor caused the release of pro-inflammatory mediators or a decrease of trans-epithelial electrical resistance in the reconstituted human epidermis (Mencherini et al., 2009). Ethanolic extracts from the leaves of *Melissa officinalis* were shown to inhibit acetic acid-induced visceral pain in a dose dependent manner with an ID_50_ value of 241.9 mg/kg as well as early (neurogenic pain) and late (inflammatory pain) phases of formalin-induced licking and glutamate-induced pain (ID_50_=198.5 mg/kg, rosmarinic acid ID_50_=2.64 mg/kg) (Guginski et al., 2009). Furthermore, rosmarinic acid from *Melissa officinalis* was shown to inhibit several complement-dependent inflammatory processes on both the classical pathway C3-convertase and on the cobra venom factor-induced, alternative pathway convertase *in vitro* (max. inhibition of classical pathway lysis observed at 2.6 mM). Moreover, significant inhibition of lysis of pre-formed EA43b cells by dilutions of human or rabbit serum in the presence of rosmarinic acid (1 mM) together with an inhibition of C5a generation was reported (Peake et al., 1991).

#### 3.1.20 Melampyrum pratense

The extracts prepared from *Melampyrum pratense* herb revealed high potency in the TNF-α and LPS-induced E-selectin and IL-8 mRNA reduction assays. Further investigations of this drug led to the isolation of iridoids, flavonoids and the phenolic compound lunularin. The flavonoids apigenin and luteolin were found to reduce TNF-α-induced NF-κB activation as well as TNF-α- and LPS-induced E-selectin and IL-8 mRNA in endothelial cells. Lunularin effectively downregulated LPS stimulated IL-8 and E-selectin and the iridoids melampyroside and mussaenoside inhibited E-selectin in LPS-stimulated cells (Vogl et al. 2013).

#### 3.1.21 Origanum vulgare

In the present study, moderate activation of PPAR-α was reached by the detannified methanolic extract of *Origanum vulgare**,*** while NF-κB activation was inhibited by its DCM extract. This plant species has been reported to possess anti-inflammatory activities *in vitro* and *in vivo.* Possible anti-inflammatory constituents were identified as rosmarinic acid, oleanolic acid and ursolic acid (Shen et al., 2010). Supercritical fluid extracts of the herb from oregano with the main compounds, trans-sabinene hydrate, thymol and carvacrol were found to decrease pro-inflammatory TNF-α, IL-1β and IL-6 cytokines and to increase the production of the anti-inflammatory cytokine IL-10 in oxidized-LDL-activated THP-1 macrophages (Ocana-Fuentes et al., 2010). During a screening of 34 dietary plants on their ability to induce basal NF-κB activity or inhibit LPS-induced NF-κB activity in monocytes stably transfected with a NF-κB-luciferase reporter construct, apigenin (present in oregano and onion), carnosol (rosemary, thyme) and sulforaphane (cruciferous vegetables) were identified as the most potent NF-κB inhibitors. The spices, oregano, thyme, clove and turmeric exhibited strong inhibition of LPS-induced NF-κB activity (less than 15% compared to control with *p*<0.001); inhibition of TNF-α-induced activation was found to be even stronger (Paur et al., 2008).

#### 3.1.22 Petasites hybridus

The present study suggests an influence of *Petasites hybridus* leaf extracts on the pro-inflammatory mediators IL-8 and E-selectin *in vitro*. Actually, this plant is mainly associated with toxicity. However, recent studies indicated that there were no signs of hepatocellular toxicity at estimated therapeutic *C*_max_ levels of 60 ng/mL. Nonetheless, when investigating liver function *in vitro* at >170-fold of therapeutic *C*_max_ levels, including cytotoxicity (measured by conversion of tetrazolium bromide (MTT) to MTT formazan, intracellular ATP using ATP bioluminescence and lactate dehydrogenase (LDH) activity), transaminase activities (alanine aminotransferase and aspartate aminotransferase), albumin synthesis, urea and testosterone metabolism to assay for cytochrome P450 monooxygenase activity, only rhizome extracts rich in petasin (37% petasin) evoked liver toxicity (Anderson et al., 2009). Lipophilic extracts of *Petasites hybridus* rhizomes with different content of petasin and isopetasin were investigated for an inhibition of COX-1 and -2 isoenzymes, whereas inhibition of the expression of COX-2 and p42/44 MAP kinase was tested in rat primary microglial cells. All extracts were weak direct inhibitors of COX-1 (IC_50_>400 μg/mL) but strong inhibitors of the inducible isoform COX-2 (IC_50_=20.0−60.6 μg/mL). As pure petasin and isopetasin neither inhibited COX-1 nor COX-2, this activity was not correlated to the content of these compounds. Moreover, the extracts inhibited LPS-induced and thus COX-2-mediated PGE2 release in primary rat microglial cells (IC_50_=2.4−5.8 μg/mL) (Fiebich et al., 2005). *In vitro* studies suggested that an extract of *Petasite hybridus* (Ze339), with petasins considered to be the pharmacologically active compounds, blocks leukotriene synthesis in monocytes and granulocytes (Thomet et al., 2001). Furthermore, this extract was reported to inhibit the allergic response via reduction of airway hyperresponsiveness and eosinophil recruitment into the bronchoalveolar lavage fluid upon allergen challenge in mice. These effects were associated with reduced IL-4, IL-5 and RANTES (chemokine (C–C motif) ligand 5) production in the bronchoalveolar lavage (Brattstrom et al., 2010). However, these findings could not be confirmed in a clinical trial (Jackson Catherine et al., 2004).

#### 3.1.23 Peucedanum ostruthium

In the present study, the detannified MeOH extracts prepared from rhizomes and leaves of *Peucedanum ostruthium* were potent inhibitors of NF-κB and showed a strong activation of PPARs. The DCM extract, on the other hand, was active on IL-8, E-selectin and NF-κB. The DCM root extract inhibited serum-induced vascular smooth muscle cell proliferation concentration-dependently with the coumarin ostruthin as the major antiproliferative substance (Joa et al., 2011; Vogl et al., 2011). Several compounds derivatized from osthole, isolated from *Peucedanum ostruthium* roots*,* showed moderate inhibitory activity in the humoral immune response to sheep erythrocytes in mice as well as on concanavalin A- and pokeweed mitogen-induced mouse splenocyte proliferation. Moreover, three compounds inhibited TNF-α in rat and human blood cells, whereas one derivative stimulated the production of both cytokines. Stimulation of IL-6 as well as a suppression of the carrageenan-induced inflammation in mice (56.5% and 68.3% inhibition, respectively) was observed by two substances (Zimecki et al., 2009). Extracts from the roots of *Peucedanum ostruthium* were investigated *in vivo* for their antiphlogistic and antipyretic effects as well as for their influence on arachidonic acid metabolism. Thereby, oral administration of 10% ethanolic extract led to a marked inhibition of carrageenan-induced rat paw edema. Here, 6-(3-carboxybut-2-enyl)-7-hydroxycoumarin was determined to be the anti-inflammatory principle of the plant (inhibition of≤50% at 30 μg/kg). Furthermore, the extract and the coumarin were characterized as dual inhibitors of COX and 5-LOX activities (Hiermann and Schantl, 1998).

#### 3.1.24 Picea abies

Concerning *Picea abies* shoot tip extracts we observed a moderate inhibition of NF-κB and weak downregulation of TNF-α induced production of E-selectin mRNA. In literature, the lignan 7-hydroxymatairesinol extracted from the heartwood of *Picea abies* was reported to reduce LPS-stimulated TNF-α secretion in THP-1 cells in a dose-dependent manner as well as TNF-α mRNA. In human polymorphonuclear leukocytes this compound concentration dependently reduced ROS and IL-8 production (Cosentino et al., 2010). On the other hand, Maeaettae et al. (2006) showed that hardwood and softwood dusts induced TNF-α, CCL2, CCL3, CCL4 and CXCL2/3 chemokine expression and inhibited IL-1β and CCL24 expression in RAW 264.7 cells (Maeaettae et al., 2006).

#### 3.1.25 Plantago lanceolata

In the present analyses, extracts from *Plantago lanceolata* leaves were able to inhibit NF-κB and to activate PPARs. Recently, Beara et al. (2012) studied the content of 44 phenolics in methanol extracts of *Plantago altissima* and *Plantago lanceolata* using LC–MS/MS. The main components in both species were *p*-hydroxybenzoic, vanillic, gallic and chlorogenic acid, apigenin, luteolin and luteolin-7-*O*-glucoside. Antioxidant activity comparable to butylated hydroxytoluene was shown in several assays. Investigations of the anti-inflammatory potential revealed that COX-1 and 12-LOX inhibition by *Plantago altissima* (IC_50_=4.4 and 3.6 mg/mL, resp.) was weaker than that of *Plantago lanceolata* (IC_50_=2.0 and 0.8 mg/mL, resp.). Moreover, cell growth was inhibited by *Plantago lanceolata* in four cell lines (IC_50_=172.3, 142.8, 405.5 and 551.7 μg/mL for HeLa, MCF7, HT-29 and MRC-5 cell lines, resp.) (Beara et al., 2012). In 2010, the same group reported a COX-1 inhibitory activity of *Plantago lanceolata* and *Plantago major* methanolic leaf extracts with an IC_50_ of 2.00 and 0.65 mg/mL, respectively, as well as an inhibition of 12-LOX (IC_50_=0.75 and 1.73 mg/mL) (Beara et al., 2010). The herbal phenylethanoid acteoside isolated from *Plantago lanceolata* leaves was reported to have anti-oxidative properties and to significantly ameliorate colitis induced by dextran sulfate sodium in Balb/c mice. T cells isolated from mesenteric lymph nodes were stimulated with anti-CD3 antibody in the presence of IL-2; after incubation for 24 h, IL-1β, IL-6, IL-12, TNF-α and IFN-γ levels in supernatants were analyzed and a significant down-regulation of IFN-γ secretion (195 pg/mL with 600 μg acteoside *vs.* 612 pg/mL with PBS, *p*<0.02) was observed (Hausmann et al., 2007). In the modified hens' egg chorioallantoic membrane test, *Plantago lanceolata* fluid extracts were comparable to hydrocortisone, phenylbutazone, and diclofenac-Na regarding their anti-inflammatory effects (Marchesan et al., 1998). Phenylethanoids, acteoside and plantamajoside isolated from the herb of *Plantago lanceolata* showed inhibitory effects on arachidonic acid-induced mouse ear edema (Murai et al., 1995). Moreover, a hydroalcoholic extract from this plant species was reported to suppress 5-LOX and COX-2 in cell-free systems (Herold et al., 2003).

#### 3.1.26 Prunella vulgaris

In the present study, we observed a strong inhibition of NF-κB along with an activation of PPAR and downregulation of E-selectin by *Prunella vulgaris* herb extracts. Hwang et al. (2012) reported that aqueous extracts of this plant species decreased high glucose-induced expression of ICAM-1, VCAM-1 and E-selectin, suppressed adhesion of HL-60 monocytic cells, secreted gelatinases as well as the formation of intracellular ROS. Additionally, as shown in our assays, inhibition of NF-κB (p65) activation in high glucose treated cells was demonstrated. Moreover they observed an activation of Akt phosphorylation, heme oxygenase-1 (HO-1), eNOS and nuclear factor E2-related factor 2 (Nrf2) (Hwang et al., 2012). Ethanolic extracts of *Prunella vulgaris* herb were shown to significantly inhibit LPS-stimulated prostaglandin E2 (PGE2) and nitric oxide production in RAW 264.7 mouse macrophages at 30 μg/mL without affecting cell viability. This effect could only be partly explained by an effect of rosmarinic acid. LPS-induced COX-2 and iNOS protein expression were reduced by *Prunella vulgaris* extract whereas rosmarinic acid inhibited only COX-2 expression (Huang et al., 2009). Moreover, *Prunella vulgaris* extracts as well as rosmarinic acid reduced ROS production, intracellular glutathione depletion as well as lipid peroxidation in LPS-treated cells. Furthermore, inhibition of LPS-induced upregulation of IL-1β, IL-6 and TNF-α and suppression of iNOS was observed (Zdarilova et al., 2009). Extracts of this plant species were reported to suppress phorbol 12-myristate 13-acetate- and calcium ionophore A23187-stimulated TNF-α, IL-6 and IL-8 secretion in human mast cells and inhibited A23187-induced NF-κB DNA binding activity as well as NF-κB-dependent reporter gene assay (Kim et al., 2007).

#### 3.1.27 Ribes nigrum

In contrast to published results, we could not observe an effect of black currant (*Ribes nigrum*) fruit extracts on NF-κB. However, activation of PPARs and reduction in stimulated IL-8 and E-selectin mRNA production was detected. Several *in vitro* and *in vivo* studies indicate that anthocyanins and other polyphenols in berries have a range of potential health benefits including anti-oxidant, anti-inflammatory, cytoprotective and neurological effects (Beattie et al., 2005; Karjalainen et al., 2009). Kivimaeki et al. (2012) studied vascular anti-inflammatory effects of cranberry (*Vaccinium oxycoccos*), lingonberry (*Vaccinium vitis-idaea*) and black currant (*Ribes nigrum*) juices given as drinking fluid *ad libitum* to spontaneously hypertensive rats. The mRNA expression of angiotensin-converting enzyme 1 (ACE1), COX-2, MCP-1 and P-selectin was significantly reduced in the cranberry and lingonberry groups (Kivimaeki et al., 2012). In a study conducted by Hurst et al. (2010), a proanthocyanin-enriched black currant fruit extract in contrast to an anthocyanin-enriched extract suppressed both IL-4- and IL-13-stimulated eotaxin-3 (CCL26) secretion in a dose-dependent manner. IL-4-stimulated CCL26 secretion was also decreased by epigallocatechin and to a lesser extent by epicatechin, metabolites identified in the proanthocyanidin extract. Moreover, epigallocatechin was able to reduce the phosphorylation of STAT-6 and to potentiate the ability of IFN-γ to suppress IL-4-stimulated CCL26 secretion (Hurst et al., 2010). Furthermore, black currant fruit extracts induced NF-κB activity in a screening of dietary plants in monocytes stably transfected with a NF-κB-luciferase reporter construct (Paur et al., 2008). Karlsen et al. (2007) investigated the effect of anthocyanins isolated from bilberries and black currants in cultured monocytes. Thereby, these compounds were found to efficiently suppress LPS-induced activation of NF-κB. In a clinical trial, differences were observed regarding the pro-inflammatory chemokines IL-8, RANTES and the cytokine IFN-α (an inducer of NF-κB activation). Hence, changes in the anthocyan supplementation group (45%, 15%, and 40% decreases from baseline, resp.) differed from those in the placebo group (20%, 0%, and 15% decreases from baseline, resp.). Similarly, changes in the pro-inflammatory cytokines IL-4 and IL-13, in the anthocyan supplementation group (60% and 38% decreases from baseline, resp.) tended to differ from those in the placebo group (4% and 6% decreases) (Karlsen et al., 2007). Proanthocyanidins isolated from *Ribes nigrum* leaves were reported to inhibit carrageenan-induced pleurisy in rats by reducing pleural exudate formation and polymorphonuclear neutrophil infiltration. Moreover, immunohistochemical investigations on lung sections showed a reduced production of endothelial cell adhesion molecules. *In vitro,* this was validated by an observed inhibition of ICAM-1 but not of IL-8 and VEGF165 mRNA expression (Garbacki et al., 2005). Prodelphinidins, from *Ribes nigrum* leaves, decreased PGE2 level and selectively inhibited COX-2 *in vitro*. In the whole blood assay though, no effects on COX activity could be found (Garbacki et al., 2002).

#### 3.1.28 Rosa canina

We detected a moderate activation of PPARs in the detannified MeOH extract of *Rosa canina* fruits, which has not been investigated so far. *Rosa canina* fruits (rose hip) were reported to contain antioxidant nutrients and an anti-inflammatory galactolipid (Kirkeskov et al., 2011). DCM extracts from rose hip powder and the triterpene acids oleanolic acid and ursolic acid (IC_50_ 21±6 μM) isolated thereof significantly and concentration dependently inhibited LPS-induced IL-6 release from Mono Mac 6 cells (Saaby et al., 2011). Rose hip powder extracts of different polarity were tested for inhibition of COX-1, COX-2, and 5-LOX-mediated leukotriene B4 (LTB4) formation as well as for DPPH-radical-scavenging capacity. In contrast to ineffective water and methanol extracts, *n*-hexane and dichloromethane extracts were potent inhibitors of COX-1, COX-2, and 5-LOX-mediated leukotriene B4. The triterpene acids ursolic acid, oleanolic acid and betulinic acid, as well as the fatty acids oleic, linoleic, and α-linolenic acid were identified in the active extracts. The methanolic extract was the most potent DPPH radical scavenger well correlated with its total phenolic content (Wenzig et al., 2008). *In vivo, Rosa canina* fruit extract inhibited the development of carrageenan-induced edema similar to indomethacin (Lattanzio et al., 2011).

#### 3.1.29 Salvia officinalis

In the present study, PPARγ activation by *Salvia officinalis* leaf extracts, as published by Poeckel et al. (2008), could not be observed. However, most extracts were strong inhibitors of NF-κB. Rodrigues et al. (2012) studied anti-inflammatory and antinociceptive effects of a hydroalcoholic extract prepared from *Salvia officinalis* leaves. The extract as well as compounds isolated thereof were tested *in vivo* in mice models. Total leukocytes, plasma extravasation as well as writhings, induced by acetic acid, were reduced after oral administration of the extract (10, 30 and 100 mg/kg). With the hydroalcoholic *Salvia officinalis* extract amelioration of neurogenic and inflammatory phases, affected by naloxone, was observed in the formalin test. Moreover, the extract was able to reduce glutamate-, capsaicin-, and cinnamaldehyde-induced nociception and paw edema at doses not affecting the locomotor activity of mice in the open field test. From the compounds studied carnosol (10 mg/kg) and ursolic acid/oleanolic acid (30 mg/kg) repressed the inflammatory phase induced by formalin and the nociception and mechanical allodynia induced by cinnamaldehyde (Rodrigues et al., 2012). Furthermore, sage was reported to have similar anti-oxidant potentials as synthetic antioxidants such as butylated hydroxytoluene and butylated hydroxylanisole, currently used as food additives (Krishnaiah et al., 2011). Moreover, extracts from sage were able to decrease pro-inflammatory IL-6 and TNF-α release. Simultaneously, an increase of the anti-inflammatory IL-10 and reduction of COX-2 or iNOS expression have been observed (Mueller et al., 2010). The phenolic diterpenes carnosic acid and carnosol, present in several labiate herbs, like *Rosmarinus officinalis* (Rosemary) and *Salvia officinalis*, were found to activate PPARγ and to inhibit the formation of pro-inflammatory leukotrienes in intact human polymorphonuclear leukocytes (IC_50_=15−20 μM and 7 μM, resp.) as well as the activity of purified recombinant 5-LOX (IC_50_=1 μM and 0.1 μM, resp.) (Poeckel et al., 2008). Moreover, hexane and ethyl acetate fractions of sage were shown to inhibit the protein and mRNA expression of TNF-α and IL-6 (at 100 μg/mL) as well as nitrite accumulation and the LPS/IFN-γ induced iNOS protein (50 µg/mL) in LPS-stimulated RAW 264.7 cells (Hyun et al., 2004). *In vivo* aqueous and butanol leaf extracts caused an analgesic effect in the hot-plate latency assay as well as in early and late phases of formalin-induced paw licking in rats (Qnais Esam et al., 2010). The chloroform extract and its main component ursolic acid (ID_50_=0.14 μM/cm^2^-2-fold more potent than indomethacin) were investigated for topical anti-inflammatory properties (Baricevic et al., 2001).

#### 3.1.30 Sambucus ebulus

In our study, *Sambucus ebulus* fruit extracts were able to reduce the production of either IL-8 or E-selectin after stimulation with TNF-α and LPS. In literature, extracts from the leaves of *Sambucus ebulus* and ursolic acid isolated thereof reduced TNF-α-induced expression of VCAM-1 (IC_50_=6.25 μM); ursolic acid additionally inhibited ICAM-1 (IC_50_=3.13−6.25 μM) (Schwaiger et al., 2011). Aqueous extracts from *Sambucus ebulus* fruit revealed NO scavenging activity (Ebrahimzadeh et al., 2010). In a review of Shokrzadeh and Saeedi Saravi (2010), anti-inflammatory, anti-nociceptive, anti-cancer, anti-angiogenic and anti-oxidative activities were described for this plant species. Thereby, several compounds including ebulitin, ebulin 1, flavonoids, and athocyanins among other components were identified as active ingredients (Shokrzadeh and Saeedi Saravi, 2010). Anti-inflammatory *in vivo* activity associated with the content of chlorogenic acid was claimed for the models carrageenan- and serotonin-induced hind paw edema in mice, and adjuvant-induced chronic arthritis in rats (Yesilada, 1998).

We demonstrated detannified MeOH extracts of *Sambucus nigra* flowers and fruits to be strong activators of PPARs and potent inhibitors of NF-κB. Barak et al. (2002) showed that the standardized black elderberry fruit extract, Sambucol, was effective against 10 strains of influenza virus *in vitro*. Moreover, the production of the inflammatory cytokines IL-1β, TNF-α, IL-6, and IL-8 was significantly increased by this extract (2–45-fold), compared to LPS, in human monocytes (Barak et al., 2001). The elder flower extract, on the other hand, was found to potently inhibit cytokine production, integrin activation, or induction of the oxidative burst via inhibition of NF-κB and phosphatidylinositol 3-kinase (Harokopakis et al., 2006).

#### 3.1.31 Sanicula europaea

Concerning *Sanicula europaea,* an *in vivo* study of its saponins injected into rats showed reduction of the inflammatory responses after injections of nucleic acid sodium salt or ovalbumin into the hindpaw (Jacker and Hiller, 1976). The present study, in addition, indicates an *in vitro* anti-inflammatory activity of *Sanicula europaea* roots due to a decreased production of IL-8 and E-selectin after stimulation with TNF-α and LPS.

#### 3.1.32 Symphytum officinale

The present analyses revealed potency of the leaf and root extracts prepared from *Symphytum officinale* to activate PPARs and to downregulate IL-8 and E-selectin mRNA. Araujo et al. (2012) evaluated the wound healing activity of comfrey leaves and 3 preparations thereof (carbomer gel, glycero-alcoholic solution and O/W emulsion/soft lotion) in an open wound rat model, using allantoin as positive control. The emulsion was found to induce the repair of damaged tissue together with an increase in collagen deposition from 40% to 240% and reduction of cellular inflammatory infiltrate by 3–46% (Araujo et al., 2012). Hiermann and Writzel (1998) reported that a glycopeptide isolated from the aqueous extract of *Symphytum officinale* roots exerted a dose-dependent antiphlogistic effect on carrageenan-induced rat paw edema with an ED_50_ of 61 μg/kg p.o. (control indomethacin 10 mg/kg). Further investigations on the release of arachidonic acid, COX and LOX metabolites as well as on arachidonic acid-induced platelet aggregation indicate that the isolated glycopeptide inhibits the release of prostaglandins and leukotrienes via a decreased expression of phospholipase A2 (Hiermann and Writzel, 1998).

#### 3.1.33 *Tilia sp.* (*Tilliae flos* in accordance with the Ph. Eur.)

Activation of PPARs by the DCM extract and moderate inhibition of NF-κB by the detannified MeOH extract of *Tilia sp.* (*Tilliae flos* in accordance with the Ph. Eur.) was detected in the present study. The flavonoids contained in the flowers of *Tilia*×*viridis* have been shown to exert antioxidant properties, acting as ROS scavengers, principally on hydrogen peroxide and the superoxide anion (Marrassini et al., 2011). *Tilia argentea* leaves and its two main flavonoid glycosides, kaempferol-3,7-*O*-α-dirhamnoside and quercetin-3,7-*O*-α-dirhamnoside, were evaluated for an antinociceptive activity, using the *p*-benzoquinone-induced writhing test and for an anti-inflammatory activity the carrageenan-induced hind paw edema model in mice. Both compounds had an activity at a dose of 50 mg/kg, per os, without inducing any apparent acute toxicity or gastric damage (Toker et al., 2004).

#### 3.1.34 Tussilago farfara

We could detect an activation of PPARs and an inhibition of NF-κB by extracts of *Tussilago farfara* leaves. Recently three new sesquiterpenoids, 1α-(3″-ethyl-cis-crotonoyloxy)-8-angeloyloxy-3β,4β-epoxy-bisabola-7,10-diene, 7β-angeloyloxy-14-hydroxy-notonipetranone and 1α-hydroxy-7β- (4-methylsenecioyloxy)- oplopa-3(14)Z,8(10)-dien-2-one isolated from an ethanolic extract of *Tussilago farfara* flower buds, along with nine known sesquiterpenoids were evaluated for an inhibition of NO release induced by LPS in RAW 264.7 macrophages. All components exhibited inhibitory activity on NO production in a dose-dependent manner with 7β-(4-methylsenecioyloxy)-oplopa-3(14)E,8(10)-dien-2-one being the most potent one (IC_50_=10.80 μM) (Li et al., 2012). Ethyl acetate extracts of *Tussilago farfara* leaves inhibited the production of NO, TNF-α, and IL-6, tested by the Griess assay and ELISA, respectively, as well as the LPS-induced phosphorylation of the NF-κB subunit p65, in RAW 264.7 cells (Xu et al., 2011). Moreover, tussilagone, a sesquiterpenoid isolated from the flower buds of *Tussilago farfara,* inhibited the production of NO, TNF-α, and PGE2 as well as iNOS and COX-2 expression in LPS-stimulated RAW 264.7 cells and murine peritoneal macrophages (Hwangbo et al., 2009).

#### 3.1.35 Urtica dioica

Our findings concerning an inhibition of NF-κB by *Urtica dioica* leaf extracts support published results from Riehmann et al. (1999) together with an activation of PPARs. Nettle leaf extracts potently inhibited NF-κB activation in various cell types and in response to several stimuli. This effect was not mediated by a direct modification of DNA binding, but rather by preventing degradation of its inhibitory subunit IκB-α (Riehemann et al., 1999). Moreover, extracts from *Urtica dioica* leaves have revealed antagonistic activity against the histamine-1 receptor (IC_50_=193±71 μg/mL) as well as reduction of prostaglandin formation through inhibition of COX-1 (IC_50_=1160±47 μg/mL), COX-2 (IC_50_=275±9 μg/mL) and hematopoietic prostaglandin D2 synthase (295±51 μg/mL), central enzymes in pro-inflammatory pathways (Roschek et al., 2009). Due to a review on this plant species several clinical and experimental studies suggest that nettle herb has some anti-inflammatory properties (Chrubasik et al., 2007). A polysaccharide fraction isolated from a water extract of nettle roots showed activity in the carrageenan rat paw edema model and lymphocyte transformation test (Wagner et al., 1989).

#### 3.1.36 *Vaccinium myrtillus* and *Vaccinium vitis-idaea*

The present investigations showed that extracts from *Vaccinium myrtillus* and *Vaccinium vitis-idaea* fruits revealed potency to inhibit NF-κB, activate PPAR and suppress the expression of IL-8 and E-selectin mRNA. Miyake et al. (2012) evaluated an anthocyanin-rich bilberry (*Vaccinium myrtillus*) extract in a mouse model of endotoxin-induced uveitis showing retinal inflammation, as well as uveitis. Pre-treatment with the extract was shown to prevent the impairment of photoreceptor cell function, measured by electroretinogram, and it decreased the production of rhodopsin and inhibited STAT3 activation, together with an increase in IL-6 expression. Moreover, it was able to reduce the intracellular release of ROS and NF-κB activation, supporting our findings (Miyake et al., 2012). Piberger et al. (2011) showed that bilberry extracts and anthocyanins thereof ameliorated acute colitis as well as chronic colitis in mice. Bilberries were reported to contain approx. 10% of anthocyanins with claimed anti-oxidative, anti-carcinogenic, and anti-inflammatory properties. When inducing acute and chronic dextran sodium sulfate (DSS) colitis in Balb/c mice by 2.5% DSS in the drinking water, oral administration of bilberries or anthocyanins thereof ameliorated disease severity and reduced secretion of IFN-γ and TNF-α from mesenteric lymph node cells measured by ELISA (Piberger et al., 2011). This may evoke the downregulation of IL-8 and E-selectin mRNA after stimulation with TNF-α and LPS as shown in our study. Moreover, treatment with extracts from *Vaccinium myrtillus* fruits were reported to ameliorate the 2,4,6-trinitro-1-chlorobenzene induced scratching behavior, and ear swelling in a mouse model of chronic allergic contact dermatitis (Yamaura et al., 2011). Additionally, this plant was able to reduce ROS level in HT-29 (250 μg/mL; 24 h, *p*<0.05) and Caco-2 (50 μg/mL; 1 h, *p*<0.05) cells (Kraus et al., 2010). In a clinical trial, supplementation with *Vaccinium myrtillus* juice resulted in significant decreases in plasma concentrations of C-reactive protein (CRP), IL-6, IL-15 and monokine induced by INF-γ (MIG) whereas, an increase in TNF-α was observed. Furthermore, the polyphenols quercetin, epicatechin, and resveratrol from bilberries inhibited LPS-induced NF-κB activation in a monocytic cell line (Karlsen et al., 2010), again supporting our findings. The anthocyanidins delphinidin 3-*O*-glucoside and delphinidin 3-*O*-galactoside were found to be the most effective soybean LOX-1 (IC_50_ values of 0.43 and 0.49 μM) and 5-LOX (IC_50_ values of 2.15 and 6.9 μM) inhibitors (Knaup et al., 2009). In cultured monocytes, anthocyanins isolated from bilberries and black currants efficiently suppressed LPS-induced activation of NF-κB. In a clinical trial, changes regarding the NF-κB-controlled pro-inflammatory chemokines IL-8, RANTES and the cytokine IFN-α in the anthocyanin-supplemented group (45%, 15%, and 40% decreases from baseline, resp.) differed from those in the placebo group (20%, 0%, and 15% decreases from baseline, resp.) (*p*<0.050). Similarly, changes in IL-4 and IL-13 in the anthocyanin-supplemented group (60% and 38% decreases from baseline, resp.) varied compared to those in the placebo group (4% and 6% decreases) (*p*=0.056 and *p*=0.089, resp.) (Karlsen et al., 2007). Phenolics from *Vaccinium vitis-idaea* fruits were only shown to suppress IL-6 and TNF-α production at a concentration of 100 μg/mL but had no significant effect on IL-1β production (Kylli et al., 2011). Moreover, extracts from *Vaccinium vitis-idaea* berries inhibited platelet activating factor-induced exocytosis *in vitro* (Tunon et al., 1995).

#### 3.1.37 *Verbascum sp.* (*Verbasci flos* in accordance with the Ph. Eur.)

The recent study revealed an activity of the DCM extract of *Verbascum sp.* (*Verbasci flos* in accordance with the Ph. Eur.) on PPARs. Akdemir et al. (2011) claimed that *Verbascum mucronatum* flowers had anti-inflammatory, antinociceptive and wound healing activities on the carrageenan-induced hind paw edema model in mice and the incision and excision models in mice and rats. Within this study four iridoid glucosides, ajugol, aucubin, lasianthoside I, catalpol, two saponins, ilwensisaponin A and C and a phenylethanoid glycoside, verbascoside were isolated. Verbascoside revealed significant wound healing activity as well as antinociceptive and anti-inflammatory potentials (Akdemir et al., 2011). Antioxidant activities were shown for ethanolic extracts of the aerial parts of *Verbascum xanthophoeniceum*, collected phenylethanoid glycosides fractions and forsythoside B, verbascoside and leucosceptoside B in the DPPH, oxygen radical absorbance capacity, hydroxyl radical averting capacity, ferric-reducing antioxidant power, and superoxide anion radical scavenging assays (Georgiev et al., 2011).

#### 3.1.38 Verbena officinalis

Concerning *Verbena officinalis*, nothing is currently known about an inhibition of NF-κB or suppression of IL-8 and E-selectin mRNA expression, as assessed by the present investigations. In literature, *Verbena officinalis* was reported as a moderate inhibitor (50%) of COX-1 (Lin et al., 2008). *In vivo* an anti-inflammatory activity using the carrageenan paw edema model was shown for extracts of different polarity, with the chloroform extract showing the highest activity. Thereof β-sitosterol, ursolic acid, oleanolic acid, 3-epiursolic acid, 3-epioleanolic acid and minor triterpenoids of derivatives of ursolic acid and oleanolic acid were isolated. From the MeOH extract 2 iridoid glycosides, verbenalin and hastatoside, a phenylpropanoid glycoside, verbascoside and β-sitosterol-D-glucoside were purified (Deepak and Handa, 2000).

#### 3.1.39 *Veronica sp. (commercial drug,) Veronica chamaedrys* and *Veronica officinalis*

The last genera investigated were *Veronica* species. Here, extracts of *Veronica chamaedrys* and *Veronica officinalis* revealed an activity on PPARs and on the pro-inflammatory mediators, IL-8 and E-selectin. *Veronica sp.* (commercial drug), however, was also active on NF-κB. Nothing has been found in the literature about the two *Veronica* species investigated in the context of inflammation. However, *in vivo* investigations of a methanolic extract of *Veronica anagallis-aquatica* L. aerial parts showed an inhibitory activity against carrageenan-induced hind paw edema and against *p*-benzoquinone-induced writhing in mice. Bioactivity-guided isolation led to the identification of aquaticoside A, aquaticoside B, aquaticoside C, veronicoside, catalposide, verproside, verminoside and martynoside of which verproside and catalposide were found to possess potent antinociceptive and anti-inflammatory activities per os, without inducing any apparent acute toxicity as well as gastric damage (Kuepeli et al., 2005). Harput et al. (2002) showed that the methanolic extracts of *Veronica cymbalaria, Veronica hederifolia, Veronica pectinata var. glandulosa, Veronica persica* and *Veronica polita* had an inhibitory activity on NO release in LPS-stimulated macrophages and cytotoxic activity against KB epidermoid carcinoma and B16 melanoma cells. After partition between water and CHCl_3_, water fractions significantly reduced NO production without any cytotoxicity, while CHCl_3_ fractions showed cytotoxicity. Moreover, the water fractions of the five *Veronica* species revealed radical scavenging activity in the DPPH assay (Harput et al., 2002).

## 4 Conclusion

Despite the once wide use of traditional European herbals, modern science has barely started to scientifically explore this treasure (Adams et al., 2009). When entering the term “Traditional European Medicine (TEM)” in the database of SciFinder only 11 citations are dedicated to this term. The concept “Traditional Chinese Medicine (TCM)”, on the other hand, reveals 25947 and “Ayurveda” 970 references. Three years ago Adams et al. (2009) stated that they found 7 hits for TEM, 4264 for TCM and 550 concerning Ayurveda. This shows a strong increase of interest in TCM but nearly none regarding European ethnomedicine. A reason for this phenomenon may be the avoidance of traditional ethnomedicine in Europe during the advance of Western medicine and the loss of knowledge in the medical profession concerning TEM, whereas TCM is a much more integrated part of medicine not only in the Chinese health system. Actually, in Europe nowadays TCM seems even more popular than its own traditional remedies. On the other hand, many species have been investigated not under the topic TEM, *e.g. Malva sylvestris* and *Urtica urens*.

The present investigations are the first to give an overview of several traditionally used anti-inflammatory herbal drugs from Austria and its adjacent regions. Thereby, the still existing but scarce lore about Austria's ethnomedicine, gathered in the so called VOLKSMED database, was the basis for the selection of the claimed anti-inflammatory plant species. Hence, 71 drugs from 63 plant species or genera and 28 families were screened for their *in vitro* anti-inflammatory activity, using PPARα, PPARγ and NF-κB luciferase reporter gene assays as well as RT-qPCR for assessing TNF-α- or LPS-induced E-selectin and IL-8 mRNA level. In order to enhance the possibility for finding new leads, those drugs were selected with a good score in the VOLKSMED database (many entries concerning the anti-inflammatory traditional use), on the one hand and a lack of relevant scientific literature, on the other hand.

Finally, multifunctional *in vitro* anti-inflammatory activities of extracts prepared from the medicinal herbs were compatible with their usage as traditional remedies against diverse inflammatory diseases. 95% of the screened extracts had high potency to activate PPARs, inhibit NF-κB activation or downregulate the inflammatory mediators, IL-8 and E-selectin *in vitro*. The drugs investigated in our study are used traditionally for centuries, which possibly indicates both *in vivo* efficacy and lack of acute toxicity. Since this work was designed as a screening study to scientifically confirm the anti-inflammatory activities of selected traditional herbal drugs, we did not perform toxicity studies. However, in each of the used assays we normalized the signal obtained from the inhibition or the activation of the investigated target with the signal obtained from internal controls. The presented results therefore indicate selective activation or inhibition of the investigated targets. While providing for first time scientific evidence for *in vitro* anti-inflammatory effectiveness of many of the investigated herbal drugs, these results at the same time point out the relevance of their further thorough investigation.

In conclusion, the present study of several traditional Austrian medicinal plants provides some evidence for the efficacy of an ethnopharmacological screening approach. However, foremost it supports the traditional application of several herbal medicines from Austria on a scientific basis.

## Figures and Tables

**Table 1:** Ethnopharmacological uses of traditional Austrian herbal drugs as documented in the VOLKSMED database.

**Scientific name**	**Part used**	**Family**	**Application form**	**Indications**	**Sample location/source**
*Agrimonia sp., Agrimoniae herba* Ph. Eur.	Herb	Rosaceae	Internal as tea	Liver and bile, gastrointestinal, respiratory tract	Kottas Pharma GmbH, Vienna, Austria
*Agropyron repens* (L.) P. Beauv.	Rhizome	Poaceae	Internal as tea, syrup or cold maceration in water, external as crude drug	Fever	Kottas Pharma GmbH, Vienna, Austria
*Ajuga genevensis* L.	Herb	Lamiaceae	Internal as tea	Respiratory tract	Laab im Walde, Lower Austria
*Ajuga reptans* L.	Herb	Lamiaceae	Internal as tea	Respiratory tract	Laab im Walde, Lower Austria
*Alnus viridis* (Chaix) DC.	Leaf	Betulaceae	Drug internal and external as tea	Fever, infections	Koenigsalm, Riedingtal, Lungau, Salzburg, Austria
*Angelica archangelica* L.	Root	Apiaceae	Internal as tea or tincture	Gastrointestinal tract, respiratory tract, nervous system, fever, infections, flu	Kottas Pharma GmbH, Vienna, Austria
*Angelica sylvestris* L.	Root	Apiaceae	Internal as tea or tincture	Gastrointestinal tract, respiratory tract, nervous system, fever, infections, flu	Alfred Richter GmbH. & CO.KG, Kufstein, Austria
*Argentina anserina* (L.) Rydb.	Herb	Rosaceae	Internal as tea	Gastrointestinal tract, gynaecology, spasm	Kottas Pharma GmbH, Vienna, Austria
*Bellis perennis* L.	Flower	Asteraceae	Internal as tea, leaves as salad	Respiratory tract, gastrointestinal	Alfred Galke GmbH, Gittelde, Germany
*Berberis vulgaris* L.	Fruit	Berberidaceae	Internal as tea, jelly or syrup	Fever, respiratory tract, infections, colds, flu	Kottas Pharma GmbH, Vienna, Austria
*Beta vulgaris* L.	Beet	Chenopodiaceae	Internal drug or juice, external compresses of drug	Respiratory tract, fever, infections	Alfred Galke GmbH, Gittelde, Germany
*Betonica officinalis* L.	Herb	Lamiaceae	Internal as tea, external as compress or bath	Gastrointestinal tract, respiratory tract, nervous system, skin, gynaecology	Neustift am Walde, Vienna Austria
*Calluna vulgaris* (L.) Hull	Herb	Ericaceae	Internal as tea	Kidney and urinary tract	Kottas Pharma GmbH, Vienna, Austria
*Capsella bursa-pastoris* (L.) Medik.	Herb	Brassicaceae	Internal as tea or tincture, external as tincture, tea, ointment, bath or powder	Locomotor system, gynaecology, cardiovascular system, hemostasis, skin	Kottas Pharma GmbH, Vienna, Austria
*Circaea lutetiana L.*	Herb	Onograceae	Internal as tea, external as cold maceration in ethanol	Rheumatism and gout, infections, fever	Sagberg, near Purkerdorf, Lower Austria
*Epilobium angustifolium* L.	Herb	Onograceae	Internal as tea	Prostate, kidney and urinary tract	Kottas Pharma GmbH, Vienna, Austria
*Epilobium montanum* L.	Herb	Onograceae	Internal as tea	Prostate, kidney and urinary tract	Hochwechsel/ Steyersberger Schwaig, Lower Austria
*Epilobium parviflorum Schreb.*	Herb	Onograceae	Internal as tea	Prostate, kidney and urinary tract	Kottas Pharma GmbH, Vienna, Austria
*Equisetum arvense* L.	Herb	Equisetaceae	Internal as tea, external as bath and compress	Kidney and urinary tract, locomotor system, rheumatism and gout, skin	Alfred Richter GmbH. & CO.KG, Kufstein, Austria
*Equisetum palustre* L.	Herb	Equisetaceae	Internal as tea, external as bath and compress	Intervertebral disks, lung cancer	Oggau, Neusiedlerseeregion, Bgld., Austria
*Euphrasia rostkoviana* Hayne	Herb	Orobanchaceae	Internal as tea, external as compress	Eyes, gastrointestinal tract	Murtal (near Powerstation Rotgüldensee) Lungau, Salzburg, Austria
*Euphrasia sp.*, *Euphrasia herba* DAC	Herb	Orobanchaceae	Internal as tea, external as compress	Eyes, gastrointestinal tract	Kottas Pharma GmbH, Vienna, Austria
*Filipendula ulmaria* (L.) Maxim.	Flower	Rosaceae	Internal as tea	Rheumatism and gout, infections, fever	Kottas Pharma GmbH, Vienna, Austria
*Filipendula ulmaria* (L.) Maxim.	Herb	Rosaceae	Internal as tea	Rheumatism and gout, infections, fever	Weißpriach, Salzburg; Gontal, near Katschberg, Carinthia, Austria
*Filipendula vulgaris* Moench	Flower	Rosaceae	Internal as tea	Rheumatism and gout, infections, fever	Neustift am Walde, Vienna Austria
*Gentiana punctata* L.	Leaf, root	Gentianaceae	Internal and external as liqueur and tea	Gastrointestinal tract, skin, locomotor system, liver and bile, paediatrics, fever, flu, rheumatism and gout	Pöllatal (east of the Grosser Hafner), Carinthia, Austria
*Glechoma hederacea* L.	Herb	Lamiaceae	Internal as salad and tea	Liver and bile, gastrointestinal tract, respiratory tract, food, kidney and urinary tract, fever, flu	Kottas Pharma GmbH, Vienna, Austria
*Geum montanum* L.	Root	Rosaceae	Internal as tea	Rheumatism and gout, infections, fever	Schattneralm, near Krakaudorf, Styria, Austria
*Geum urbanum* L.	Herb	Rosaceae	Internal as tea	Rheumatism and gout, infections, fever	Alfred Galke GmbH, Gittelde, Germany
*Geum urbanum* L.	Root	Rosaceae	Internal as tea	Rheumatism and gout, infections, fever	Alfred Galke GmbH, Gittelde, Germany
*Hippophae rhamnoides* L.	Fruit	Elaeagnaceae	Internal as tea, juice or syrup	Infections, colds, flu	Alfred Galke GmbH, Gittelde, Germany
*Hypericum maculatum* Crantz	Herb	Hypericaceae	Internal as oil and tea, external as oil, ointment or cold maceration in ethanol	Skin, locomotor system, nervous system, gastrointestinal tract, respiratory tract, kidney and urinary tract, cardiovascular system, infections, rheumatism and gout	Gontal, near Katschberg, Carinthia, Austria
*Linum usitatissimum* L.	Seeds	Linaceae	Internal soaked or as tea, external as compress or oil	Respiratory tract, eyes, infections, colds, flu, fever, rheumatism and gout	Alfred Richter GmbH. & CO.KG, Kufstein, Austria
*Lycopodium sp., Lycopodii herba*	Herb	Lycopodiaceae	External as compress, internal as tea	Locomotor system, skin, liver and bile, kidney and urinary tract, infections, rheumatism and gout	Kottas Pharma GmbH, Vienna, Austria, Saukel
*Majorana hortensis* Moench	Herb	Lamiaceae	Internal as herb	Gastrointestinal tract, infections	Alfred Richter GmbH. & CO.KG, Kufstein, Austria
*Malva neglecta*	Herb	Malvaceae	Internal as tea, external as bath	Skin, gastrointestinal tract, respiratory tract	Nodendorf, near Ernstbrunn, Lower Austria
*Malva sp., Malvae folium* Ph. Eur.	Leaf	Malvaceae	Internal as tea, external as bath	Skin, gastrointestinal tract, respiratory tract	Kottas Pharma GmbH, Vienna, Austria
*Melampyrum pratense* L.	Herb	Orobanchaceae	Internal as tea, external as pillow	Calcification blood vessels, rheumatism	Neustift am Walde, Vienna Austria
*Melissa officinalis* L.	Leaf	Lamiaceae	Internal as tea, external essential oil	Gastrointestinal tract, nervous system, liver and bile, food	Alfred Galke GmbH, Gittelde, Germany
*Origanum vulgare L.*	Herb	Lamiaceae	Internal as tea, external as ointment	Gastrointestinal tract, respiratory tract, nervous system	Alfred Richter GmbH. & CO.KG, Kufstein, Austria
*Petasites hybridus* (L.) Gaertn., B. Mey. & Scherb.	Leaf	Asteraceae	Internal as tea or cold maceration in ethanol, external as compress or maceration in vinegar	Infections, fever, flu, colds	Hohe Student, near Halltal, Styria, Austria
*Peucedanum ostruthium* (L.) W.D.J. Koch	Root	Apiaceae	Internal as tea, liqueur and wine, external as fumigation, tincture and incense	Gastrointestinal tract, skin, respiratory tract, cardiovascular system, viral infects, infections, fever, flu, colds	Kottas Pharma GmbH, Vienna, Austria
*Peucedanum ostruhtium* (L.) W.D.J. Koch	Leaf	Apiaceae	Internal as tea and liqueur, external as fumigation, tincture and incense	Gastrointestinal tract, skin, respiratory tract, cardiovascular system, viral infects, infections, fever, flu, colds	Hochwechsel/ Steyersberger Schwaig, Lower Austria
*Picea abies* (L.) H. Karst.	Shoot tip	Piceaceae	Internal as syrup, tea, external as resin, bath, inhalation, ointment, tea	Respiratory tract, skin, locomotor system, viral infects, gastrointestinal tract	Wechsel, Mariensee, Lower Austria
*Pimpinella major* (L.) Hudson	Root	Apiaceae	Internal as drug, tea, in milk, liqueur	Respiratory tract, fever, infections, colds, flu	Kienstein, Ebenwaldhöhe, near Kleinzell, Lower Austria
*Plantago lanceolata* L.	Leaf	Plantaginaceae	Internal as syrup or tea, external as fresh stumped leaves	Respiratory tract, skin, insect bites, viral infects	Kottas Pharma GmbH, Vienna, Austria
*Prunella vulgaris* L.	Herb	Lamiaceae	Internal as tea	Respiratory tract, infections	Neustift am Walde, Vienna, Austria
*Ribes nigrum* L.	Fruit	Grossulariaceae	Internal as food or syrup	Gastrointestinal tract, viral infects, locomotor system, respiratory tract, cardiovascular system	Alfred Richter GmbH. & CO.KG, Kufstein, Austria
*Rosa canina* L.	Fruit	Rosaceae	Internal as tea	Kidney and urinary tract, viral infects, food	Kottas Pharma GmbH, Vienna, Austria
*Rumex alpinus L.*	Leaf, root	Polygonaceae	Internal as drug	Viral infects	Hohe Student, near Halltal, Styria, Austria
*Salvia officinalis L.*	Herb	Lamiaceae	Internal as tea or chewed	Respiratory tract, mouth, gastrointestinal tract, skin	Alfred Richter GmbH. & CO.KG, Kufstein, Austria
*Sambucus ebulus L.*	Fruit	Adoxaceae		Fever, respiratory tract	Laab im Walde, Lower Austria
*Sambucus nigra* L.	Fruit	Adoxaceae	Internal as tea, jelly, juice or syrup	Viral infects, fever, flu, colds, respiratory tract, mouth, gastrointestinal tract, skin	Kottas Pharma GmbH, Vienna, Austria
*Sambucus nigra* L.	Flowers	Adoxaceae	Internal as tea or syrup	Viral infects, fever, flu, colds, respiratory tract, mouth, gastrointestinal tract, skin	Sagberg, near Purkersdorf, Lower Austria
*Sanicula europaea* L.	Root	Apiaceae	Internal as tea, external as ointment	Skin, respiratory tract, locomotor system, gastrointestinal tract, infections	Jubilaeumswarte, Vienna, Austria
*Sorbus aucuparia* L.	Fruit	Rosaceae	Internal as tea, syrup, jelly or liqueur	Respiratory tract, fever, infections, colds, flu, rheumatism and gout	Kottas Pharma GmbH, Vienna, Austria
*Symphytum officinale* L.	Root	Boraginaceae	Internal as tea or tincture, external as ointment, compress or alcoholic digestion	Locomotor system, gastrointestinal tract	Kottas Pharma GmbH, Vienna, Austria
*Symphytum officinale* L.	Leaf stem	Boraginaceae	External as ointment, compress, or alcoholic digestion, internal as tea or tincture	Locomotor system, gastrointestinal tract, rheumatism and gout	Oggau, Neusiedlersee- region, Bgld., Austria
*Tilia sp., Tiliae flos* Ph. Eur.	Flowers	Malvaceae	Internal as tea	Fever, flu, viral infects, respiratory tract	Kottas Pharma GmbH, Vienna, Austria
*Tussilago farfara* L.	Leaf	Asteraceae	Internal as tea or syrup, external direct or stumped	Respiratory tract, skin, locomotor system, viral infects, flu, colds, fever, rheumatism and gout	Alfred Richter GmbH. & CO.KG, Kufstein, Austria
*Urtica dioica* L.	Herb	Urticaceae	Internal as tea or fresh leaves	Kidney and urinary tract, gastrointestinal tract, locomotor system, skin, hemorrhage, cardio-vascular system, rheumatism and gout, flu	Alfred Richter GmbH. & CO.KG, Kufstein, Austria
*Vaccinium myrtillus L.*	Fruit	Ericaceae	Internal as fresh food, liqueur or tea	Gastrointestinal tract, diabetes	Alfred Richter GmbH. & CO.KG, Kufstein, Austria
*Vaccinium vitis-idaea L.*	Fruit	Ericaceae	Internal as jelly or syrup	Fever, gastrointestinal tract, kidney and urinary tract, food	Hochwechsel/ Steyersberger Schwaig, Lower Austria
*Verbascum sp.,Verbasci flos Ph. Eur.*	Flower	Scrophulariaceae	Internal as tea, external as ointment, tea, bath or compress	Respiratory tract, skin, veins, gastrointestinal tract, locomotor system	Alfred Richter GmbH. & CO.KG, Kufstein, Austria
*Verbena officinalis L.*	Herb	Verbenaceae	Internal as tea or liqueur	Fever, infections	Kottas Pharma GmbH, Vienna, Austria
*Veronica chamaedrys* L.	Herb	Plantaginaceae	Internal as tea	Nervous system, respiratory tract, cardiovascular system, metabolism	Neustift am Walde, Vienna, Austria
*Veronica officinalis* L.	Herb	Plantaginaceae	Internal as tea	Nervous system, respiratory tract, cardiovascular system, metabolism	Neustift am Walde, Vienna, Austria
*Veronica sp., Veronica herba* DAC	Herb	Plantaginaceae	Internal as tea	Nervous system, respiratory tract, cardiovascular system, metabolism	Kottas Pharma GmbH, Vienna, Austria

**Table 2:** *In vitro* screening results of extracts produced from 71 traditionally used Austrian herbal drugs on anti-inflammatory targets. Extracts were tested in triplicate at a concentration of 10 µg/mL on PPARs activation as well as on NF-κB inhibition and at 100 µg/mL concerning LPS- or TNF-α-induced downregulation of interleukine-8 (IL-8) and E-selectin mRNA;<25% inhibition/activation is considered as no activity “NA”, 25–50% inhibition/activation is interpreted as low activity “weak”, 50–75% inhibition/activation is considered as a moderate activity “moderate”, and high activity is defined as >75–100% inhibition/activation “strong”. NT—not tested, DCM—dichloromethane extract, MeOH—methanol extract, wCh—dichloromethane extract without chlorophyll, Det—detannified methanol extract.

**Sample**			**Results**						
**Species**	**Plant part**	**Extract**	**PPAR-α**	PPAR-γ	NF-κB	TNF-α induced		LPS induced	
						E-selectin	IL-8	E-selectin	IL-8
*Agrimonia sp.*	Herb	DCM	NA	NA	Moderate	NT	NT	NT	NT
		wCh	NA	NA	Strong	NT	NT	NT	NT
		MeOH	NA	NA	NA	NT	NT	NT	NT
		Det	NA	NA	NA	NT	NT	NT	NT

*Agropyron repens*	Rhizomes	DCM	Strong	Strong	NA	Strong	Moderate	Moderate	Strong
		MeOH	NA	NA	NA	Moderate	Moderate	NA	Moderate
		Det	NA	Moderate	NA	NT	NT	NT	NT

*Ajuga genevensis*	Herb	DCM	NA	NA	NA	Strong	Moderate	NA	Moderate
		wCh	NA	NA	NA	NT	NT	NT	NT
		MeOH	NA	NA	NA	Strong	NA	Strong	NA
		Det	NA	NA	NA	NT	NT	NT	NT

*Ajuga reptans*	Herb	DCM	NA	NA	NA	NA	Moderate	NA	Moderate
		wCh	NA	NA	NA	NT	NT	NT	NT
		MeOH	NA	NA	NA	Strong	NA	Strong	NA
		Det	NA	NA	NA	NT	NT	NT	NT

*Alnus viridis*	Leaves	DCM	NA	NA	Moderate	NT	NT	NT	NT
		wCh	NA	NA	Strong	NT	NT	NT	NT
		MeOH	NA	NA	NA	NT	NT	NT	NT
		Det	Moderate	Moderate	Strong	NT	NT	NT	NT

*Angelica archangelica*	Roots	DCM	NA	NA	Moderate	NT	NT	NT	NT
		MeOH	NA	NA	NA	NT	NT	NT	NT
		Det	NA	NA	NA	NT	NT	NT	NT

*Angelica sylvestris*	Roots	DCM	NA	NA	NA	Strong	Moderate	Moderate	Strong
		MeOH	NA	NA	NA	Moderate	NA	NA	Strong
		Det	Strong	Moderate	Strong	NT	NT	NT	NT

*Bellis perennis*	Flowers	DCM	Strong	Moderate	Moderate	NT	NT	NT	NT
		MeOH	NA	NA	NA	NT	NT	NT	NT
		Det	Moderate	Moderate	NA	NT	NT	NT	NT

*Berberis vulgaris*	Fruits	DCM	NA	NA	NA	NT	NT	NT	NT
		MeOH	NA	NA	NA	NT	NT	NT	NT
		Det	Moderate	NA	NA	NT	NT	NT	NT

*Beta vulgaris*	Roots	DCM	NA	NA	NA	NT	NT	NT	NT
		MeOH	NA	NA	NA	NT	NT	NT	NT
		Det	NA	NA	NA	NT	NT	NT	NT

*Betonica officinalis*	Herb	DCM	NA	NA	NA	Strong	Strong	Weak	NA
		wCh	NA	NA	NA	Strong	Strong	Strong	Strong
		MeOH	NA	NA	NA	Moderate	Strong	Moderate	Strong
		Det	NA	NA	NA	Strong	Strong	Weak	Strong

*Calluna vulgaris*	Herb	DCM	NA	NA	Moderate	NT	NT	NT	NT
		wCh	NA	NA	Moderate	NT	NT	NT	NT
		MeOH	NA	NA	NA	NT	NT	NT	NT
		Det	NA	NA	NA	NT	NT	NT	NT

*Capsella bursa-pastoris*	Herb	DCM	Strong	NA	Strong	Strong	Weak	Moderate	Strong
		wCh	Strong	Moderate	NA	NT	NT	NT	NT
		MeOH	NA	NA	NA	Moderate	Moderate	NA	Strong
		Det	Strong	Strong	Strong	NT	NT	NT	NT

*Circaea lutetiana*	Herb	DCM	NA	NA	NA	NT	NT	NT	NT
		wCh	Moderate	NA	NA	NT	NT	NT	NT
		MeOH	NA	NA	NA	NT	NT	NT	NT
		Det	NA	NA	NA	NT	NT	NT	NT

*Epilobium angustifolium*	Herb	DCM	NA	NA	NA	NT	NT	NT	NT
		wCh	NA	NA	Moderate	NT	NT	NT	NT
		MeOH	NA	NA	NA	NT	NT	NT	NT
		Det	NA	NA	NA	NT	NT	NT	NT

*Epilobium montanum*	Herb	DCM	NA	NA	NA	moderate	NA	Moderate	Strong
		wCh	Moderate	NA	Moderate	NT	NT	NT	NT
		MeOH	NA	NA	NA	NA	NA	NA	NA
		Det	Strong	Moderate	Moderate	NT	NT	NT	NT

*Epilobium parviflorum*	Herb	DCM	NA	NA	Moderate	NT	NT	NT	NT
		wCh	NA	NA	NA	NT	NT	NT	NT
		MeOH	NA	NA	NA	NT	NT	NT	NT
		Det	NA	NA	NA	NT	NT	NT	NT

*Equisetum arvense*	Herb	DCM	NA	NA	Strong	NT	NT	NT	NT
		wCh	Moderate	Moderate	Moderate	NT	NT	NT	NT
		MeOH	NA	NA	NA	NT	NT	NT	NT
		Det	NA	NA	NA	NT	NT	NT	NT

*Equisetum palustre*	Herb	DCM	NA	NA	NA	NA	NA	NA	Moderate
		wCh	NA	NA	Moderate	NT	NT	NT	NT
		MeOH	NA	NA	NA	Strong	NA	Moderate	NA
		Det	NA	NA	NA	NT	NT	NT	NT

*Euphrasia sp.*	Herb	DCM	NA	NA	Strong	NT	NT	NT	NT
		wCh	NA	NA	NA	NT	NT	NT	NT
		MeOH	NA	NA	NA	NT	NT	NT	NT
		Det	NA	NA	Strong	NT	NT	NT	NT

*Euphrasia rostkoviana*	Herb	DCM	NA	NA	NA	NT	NT	NT	NT
		wCh	Strong	NA	Moderate	NT	NT	NT	NT
		MeOH	NA	NA	NA	NT	NT	NT	NT
		Det	Strong	Moderate	NA	NT	NT	NT	NT

*Filipendula ulmaria*	Flowers	DCM	NA	NA	NA	NT	NT	NT	NT
		wCh	Moderate	Moderate	NA	NT	NT	NT	NT
		MeOH	NA	NA	NA	NT	NT	NT	NT
		Det	NA	NA	NA	NT	NT	NT	NT

*Filipendula ulmaria*	Herb	DCM	NA	NA	Moderate	NT	NT	NT	NT
		wCh	NA	NA	Moderate	NT	NT	NT	NT
		MeOH	NA	NA	Na	NT	NT	NT	NT
		Det	NA	NA	Strong	NT	NT	NT	NT

*Filipendula vulgaris*	Flowers	DCM	NA	NA	NA	Strong	Moderate	NA	Moderate
		MeOH	NA	NA	NA	Strong	Moderate	Strong	NA
		Det	NA	NA	NA	NT	NT	NT	NT

*Gentiana punctata*	Leaves	DCM	NA	NA	NA	NT	NT	NT	NT
		wCh	NA	NA	NA	NT	NT	NT	NT
		MeOH	NA	NA	NA	NT	NT	NT	NT
		Det	NA	NA	NA	NT	NT	NT	NT

*Gentiana punctata*	Roots	DCM	NA	NA	NA	NT	NT	NT	NT
		MeOH	NA	NA	NA	NT	NT	NT	NT
		Det	Moderate	NA	NA	NT	NT	NT	NT

*Geum montanum*	Roots	DCM	NA	NA	NA	Strong	Strong	Weak	NA
		MeOH	NA	NA	NA	NA	Strong	NA	NA
		Det	Moderate	Moderate	NA	NT	NT	NT	NT

*Geum urbanum*	Herb	DCM	Moderate	Moderate	Strong	NT	NT	NT	NT
		wCh	Strong	Strong	Moderate	NT	NT	NT	NT
		MeOH	NA	NA	NA	NT	NT	NT	NT
		Det	NA	NA	Moderate	NT	NT	NT	NT

*Geum urbanum*	Roots	DCM	Moderate	NA	Moderate	NA	Strong	Moderate	NA
		MeOH	NA	NA	NA	NA	Strong	NA	NA
		Det	Strong	Moderate	Moderate	NT	NT	NT	NT

*Glechoma hederacea*	Herb	DCM	NA	NA	NA	NT	NT	NT	NT
		wCh	NA	Moderate	Moderate	NT	NT	NT	NT
		MeOH	NA	NA	NA	NT	NT	NT	NT
		Det	Strong	Strong	Strong	Strong	Strong	NA	NA

*Hippophae rhamNAides*	Fruits	DCM	NA	NA	NA	NT	NT	NT	NT
		MeOH	NA	NA	NA	NT	NT	NT	NT
		Det	NA	NA	NA	NT	NT	NT	NT

*Hypericum maculatum*	Herb	DCM	NA	Moderate	Moderate	NT	NT	NT	NT
		wCh	Moderate	NA	NA	NT	NT	NT	NT
		MeOH	NA	NA	NA	NT	NT	NT	NT
		Det	Moderate	NA	NA	NT	NT	NT	NT

*Linum usitatissimum*	Seeds	DCM	NA	NA	NA	NT	NT	NT	NT
		MeOH	NA	NA	NA	NT	NT	NT	NT
		Det	NA	NA	NA	NT	NT	NT	NT

*Lycopodium sp.*	Herb	DCM	NA	NA	Strong	NT	NT	NT	NT
		wCh	NA	NA	Strong	NT	NT	NT	NT
		MeOH	NA	NA	NA	NT	NT	NT	NT
		Det	Moderate	Moderate	NA	NT	NT	NT	NT

*Majorana hortensis*	Herb	DCM	NA	NA	NA	NT	NT	NT	NT
		wCh	NA	NA	Strong	NT	NT	NT	NT
		MeOH	NA	NA	NA	NT	NT	NT	NT
		Det	Strong	Moderate	Moderate	NT	NT	NT	NT

*Malva neglecta*	Herb	DCM	NA	NA	NA	Strong	NA	Weak	Strong
		wCh	Strong	Moderate	NA	NT	NT	NT	NT
		MeOH	NA	NA	NA	NA	NA	NA	Strong
		Det	Moderate	NA	Moderate	NT	NT	NT	NT

*Malva sp.*	Leaves	DCM	Moderate	NA	Strong	NT	NT	NT	NT
		wCh	Strong	Strong	NA	Strong	Moderate	NA	NA
		MeOH	NA	NA	NA	NT	NT	NT	NT
		Det	NA	Strong	Moderate	NT	NT	NT	NT

*Melampyrum pratense*	Herb	DCM	NA	NA	NA	Strong	Weak	Moderate	Strong
		wCh	NA	NA	NA	Moderate	Strong	Moderate	Strong
		MeOH	NA	NA	NA	Strong	Strong	Strong	Strong
		Det	NA	NA	Strong	Strong	Strong	Moderate	Strong

*Melissa officinalis*	Leaves	DCM	NA	NA	Moderate	NT	NT	NT	NT
		wCh	Strong	Strong	Moderate	NT	NT	NT	NT
		MeOH	NA	NA	NA	NT	NT	NT	NT
		Det	NA	NA	Strong	NT	NT	NT	NT

*Origanum vulgare*	Herb	DCM	NA	NA	Moderate	NT	NT	NT	NT
		wCh	NA	NA	NA	NT	NT	NT	NT
		MeOH	NA	NA	NA	NT	NT	NT	NT
		Det	Moderate	NA	NA	NT	NT	NT	NT

*Petasites hybridus*	Leaves	DCM	NA	NA	NA	Strong	Moderate	Moderate	Moderate
		wCh	NA	NA	NA	NT	NT	NT	NT
		MeOH	NA	NA	NA	NA	Strong	NA	Moderate
		Det	NA	NA	Moderate	NT	NT	NT	NT

*Peucedanum ostruthium*	Leaves	DCM	NA	NA	NA	Strong	Strong	NA	NA
		wCh	NA	NA	NA	NT	NT	NT	NT
		MeOH	NA	NA	NA	NT	NT	NT	NT
		Det	Moderate	Strong	Moderate	NA	Weak	NA	Strong

*Peucedanum ostruthium*	Roots	DCM	NA	NA	Strong	NT	NT	NT	NT
		MeOH	NA	NA	NA	NT	NT	NT	NT
		Det	Strong	Moderate	Strong	NT	NT	NT	NT

*Picea abies*	Shoot tip	DCM	NA	NA	Moderate	NA	NA	NA	NA
		wCh	NA	NA	NA	NT	NT	NT	NT
		MeOH	NA	NA	NA	Weak	NA	NA	NA
		Det	NA	NA	NA	NT	NT	NT	NT

*Pimpinella major*	Roots	DCM	NA	NA	Moderate	NA	Strong	NA	NA
		MeOH	NA	NA	NA	NA	Moderate	NA	NA
		Det	NA	NA	Moderate	NT	NT	NT	NT

*Plantago lanceolata*	Leaves	DCM	NA	NA	Strong	NT	NT	NT	NT
		wCh	NA	Moderate	Strong	NT	NT	NT	NT
		MeOH	NA	NA	NA	NT	NT	NT	NT
		Det	Strong	Moderate	Moderate	NT	NT	NT	NT

*Potentilla anserina*	Herb	DCM	NA	NA	Strong	NT	NT	NT	NT
		wCh	NA	NA	NA	NT	NT	NT	NT
		MeOH	NA	NA	NA	NT	NT	NT	NT
		Det	NA	Moderate	NA	NT	NT	NT	NT

*Prunella vulgaris*	Herb	DCM	NA	NA	NA	Strong	NA	NA	NA
		wCh	Moderate	Moderate	Strong	NT	NT	NT	NT
		MeOH	NA	NA	NA	Strong	NA	Moderate	NA
		Det	NA	NA	NA	NT	NT	NT	NT

*Ribes nigrum*	Fruits	DCM	Moderate	Moderate	NA	Moderate	Strong	NA	NA
		MeOH	NA	NA	NA	NT	NT	NT	NT
		Det	Strong	Moderate	NA	NA	Strong	NA	Moderate

*Rosa canina*	Fruits	DCM	NA	NA	NA	NT	NT	NT	NT
		MeOH	NA	NA	NA	NT	NT	NT	NT
		Det	Moderate	Moderate	NA	NT	NT	NT	NT

*Rumex alpinus*	Leaves	DCM	NA	NA	NA	Strong	Weak	Moderate	NA
		wCh	NA	NA	NA	NT	NT	NT	NT
		MeOH	NA	NA	NA	NA	Strong	NA	Moderate
		Det	NA	NA	NA	NT	NT	NT	NT

*Rumex alpinus*	Roots	DCM	NA	NA	NA	NA	Strong	NA	NA
		MeOH	NA	NA	NA	NA	Strong	NA	Strong
		Det	NA	NA	NA	NT	NT	NT	NT

*Salvia officinalis*	Leaves	DCM	NA	NA	Strong	NT	NT	NT	NT
		wCh	NA	NA	Strong	NT	NT	NT	NT
		MeOH	NA	NA	NA	NT	NT	NT	NT
		Det	NA	NA	Strong	NT	NT	NT	NT

*Sambucus ebulus*	Fruits	DCM	NA	NA	NA	NA	Weak	Moderate	NA
		MeOH	NA	NA	NA	Strong	Strong	Weak	Moderate
		Det	NA	NA	NA	NT	NT	NT	NT

*Sambucus nigra*	Flowers	DCM	NA	NA	NA	NT	NT	NT	NT
		MeOH	NA	NA	NA	NT	NT	NT	NT
		Det	Strong	Moderate	NA	NT	NT	NT	NT

*Sambucus nigra*	Fruits	DCM	NA	NA	NA	NT	NT	NT	NT
		MeOH	NA	NA	NA	NT	NT	NT	NT
		Det	Strong	Strong	Strong	NT	NT	NT	NT

*Sanicula europaea*	Roots	DCM	NA	NA	NA	Moderate	Moderate	Strong	Strong
		MeOH	NA	NA	NA	Strong	Moderate	NA	Strong
		Det	NA	NA	NA	NT	NT	NT	NT

*Sorbus aucuparia*	Fruits	DCM	NA	NA	NA	NT	NT	NT	NT
		MeOH	NA	NA	NA	NT	NT	NT	NT
		Det	Strong	Strong	NA	NT	NT	NT	NT

*Symphytum officinale*	Stems	DCM	NA	NA	NA	Moderate	NA	Strong	Strong
		wCh	Strong	Moderate	NA	NT	NT	NT	NT
		MeOH	NA	NA	NA	NA	NA	NA	NA
		Det	Strong	Moderate	NA	NT	NT	NT	NT

*Symphytum officinale*	Leaves	DCM	NA	NA	NA	Moderate	NA	Strong	Strong
		wCh	Moderate	NA	NA	NT	NT	NT	NT
		MeOH	NA	NA	NA	Strong	NA	NA	Strong
		Det	NA	NA	NA	NT	NT	NT	NT

*Symphytum officinale*	Roots	DCM	Moderate	Moderate	NA	NT	NT	NT	NT
		MeOH	NA	NA	NA	NT	NT	NT	NT
		Det	NA	NA	NA	NT	NT	NT	NT

*Tilia sp.*	Flowers	DCM	Moderate	Moderate	NA	NT	NT	NT	NT
		MeOH	NA	NA	NA	NT	NT	NT	NT
		Det	NA	NA	Moderate	NT	NT	NT	NT

*Tussilago farfara*	Leaves	DCM	Moderate	NA	Moderate	NT	NT	NT	NT
		wCh	Strong	Moderate	Moderate	NT	NT	NT	NT
		MeOH	NA	NA	NA	NT	NT	NT	NT
		Det	Moderate	Moderate	NA	NT	NT	NT	NT

*Urtica dioica*	Leaves	DCM	Strong	NA	NA	NT	NT	NT	NT
		wCh	Strong	Strong	Moderate	NT	NT	NT	NT
		MeOH	NA	NA	NA	NT	NT	NT	NT
		Det	NA	Moderate	Moderate	NT	NT	NT	NT

*Vaccinium myrtillus*	Fruits	DCM	Moderate	Moderate	NA	NA	NA	NA	Moderate
		MeOH	NA	NA	NA	Strong	Weak	Strong	Weak
		Det	Moderate	Moderate	NA	NT	NT	NT	NT

*Vaccinium vitis-idaea*	Fruits	DCM	NA	NA	NA	NA	NA	NA	Weak
		MeOH	NA	NA	NA	NT	NT	NT	NT
		Det	Moderate	Strong	Moderate	NA	Strong	Moderate	NA

*Verbascum sp.*	Flowers	DCM	Strong	Moderate	NA	NT	NT	NT	NT
		MeOH	NA	NA	NA	NA	NA	NA	NA
		Det	NA	NA	NA	NT	NT	NT	NT

*Verbena officinalis*	Herb	DCM	NA	NA	Strong	Strong	NA	NA	Moderate
		wCh	NA	NA	NA	NT	NT	NT	NT
		MeOH	NA	NA	NA	NT	NT	NT	NT
		Det	NA	NA	Strong	NT	NT	NT	NT

*Veronica chamaedrys*	Herb	DCM	NA	NA	NA	NT	NT	NT	NT
		wCh	Strong	Moderate	NA	Moderate	Moderate	Moderate	Strong
		MeOH	NA	NA	NA	Strong	NA	NA	Strong
		Det	NA	NA	NA	NT	NT	NT	NT

*Veronica officinalis*	Herb	DCM	NA	NA	NA	Strong	Weak	Moderate	Strong
		wCh	Strong	Moderate	NA	NT	NT	NT	NT
		MeOH	NA	NA	NA	Strong	NA	NA	NA
		Det	NA	NA	NA	NT	NT	NT	NT

*Veronica sp.*	Herb	DCM	NA	NA	Strong	NT	NT	NT	NT
		wCh	Moderate	Strong	Moderate	NT	NT	NT	NT
		MeOH	NA	Strong	NA	NT	NT	NT	NT
		Det	NA	NA	NA	NT	NT	NT	NT
